# Simulation of Microbial Response to Accidental Diesel Spills in Basins Containing Brackish Sea Water and Sediment

**DOI:** 10.3389/fmicb.2020.593232

**Published:** 2020-12-23

**Authors:** Lijuan Yan, Nan Hui, Suvi Simpanen, Laura Tudeer, Martin Romantschuk

**Affiliations:** ^1^School of Agriculture and Biology, Shanghai Jiao Tong University, Shanghai, China; ^2^Faculty of Biological and Environmental Sciences, University of Helsinki, Lahti, Finland

**Keywords:** microbial community, oil contamination, littoral sediment, simulation experiment, coastal brackish water

## Abstract

The brackish Baltic Sea is under diesel oil pollution risk due to heavy ship traffic. The situation is exasperated by densely distributed marinas and a vigorous although seasonal recreational boating. The seasonality and physical environmental variations hamper the monitoring of microbial communities in response to diesel oil spills. Hence, an 8-week simulation experiment was established in metal basins (containing 265 L sea water and 18 kg quartz sand or natural shore sand as the littoral sediment) to study the effect of accidental diesel oil spills on microbial communities. Our results demonstrated that microbial communities in the surface water responded to diesel oil contamination, whereas those in the littoral sediment did not, indicating that diesel oil degradation mainly happened in the water. Diesel oil decreased the abundance of bacteria and fungi, but increased bacterial diversity in the water. Time was the predominant driver of microbial succession, attributable to the adaption strategies of microbes. Bacteria were more sensitive to diesel oil contamination than fungi and archaea. Diesel oil increased relative abundances of bacterial phyla, Alphaproteobacteria, Betaproteobacteria, Gammaproteobacteria, Flavobacteriia and Cytophagia, and fungal phylum Ascomycota in the surface water. Overall, this study improves the understanding of the immediate ecological impact of accidental diesel oil contamination, providing insights into risk management at the coastal area.

## Introduction

Diesel oil spill accidents are common in the sea- and coastal areas. For example, PAHs were often found with elevated concentrations at the coastal areas of the Baltic Sea because of human impact (Witt, 1995). Small illegal or accidental oil spills were frequently recorded in the Baltic Sea (HELCOM, 2014). Several studies determine the long-term exposure to low concentrations of oil on marine microorganisms at the coastal area of the Baltic Sea (e.g., Witt, 1995; Yan et al., 2018b). However, pollutants in a freshly contaminated environment are considered more toxic to the native microorganisms, extending the adaptation time before degradation of the pollutants commences, and even inhibiting the biodegradation (Trindade et al., 2005). Hence, the ecological response of an accidental oil spill in a short period is of great significance for assessing the ecotoxicity of contaminants.

Natural attenuation is an important process in accidental oil spill sites. Microbial communities drive many ecosystem processes, such as biogeochemical cycles of carbon, nitrogen and sulfur (Falkowski et al., 2008). Many types of microorganisms are involved in the bioremediation of oil-contaminated environments (Suni et al., 2007; Mason et al., 2012; Lamendella et al., 2014). Prokaryotes are key players in the biogeochemical cycling both as primary producers and organic matter degraders in marine environment (Massana and Logares, 2013). Microbes (e.g., bacteria and fungi) may restore marine environments after oil spills owing to their adaptation strategies and metabolic potential (Bovio et al., 2017; Varjani, 2017). Bacterial communities play important roles in degrading the saturated and partially aromatic hydrocarbons, whereas fungal communities were found to be responsible for the degradation of polar hydrocarbons comprised of recalcitrant metabolites arising from a successive degradation strategy in oil-contaminated soil (Liu et al., 2011). Considering their prevalence in terrestrial environments, relatively little attention has, however, been paid to the presence of fungi in the aquatic and sediment environments compared to other microorganisms (Pereira et al., 2010; Kumar, 2017). The existing studies on the response of oil spills on marine fungal populations were rare and mostly limited to culture-based studies, leaving the vast majority of uncultivable marine fungi (>95%) uninvestigated (Wang et al., 2012; Bovio et al., 2017; Amend et al., 2019; Gladfelter et al., 2019).

The recent development and application of high-throughput sequencing techniques have remarkably advanced our understanding of the diversity and ecology of uncultivable microbial communities in different habitats. Many well-designed 16S rDNA-based global studies have successfully characterized the biogeographic distribution patterns and diversity of uncultured prokaryotes (Lozupone and Knight, 2007; Nemergut et al., 2011; Zinger et al., 2011). Phylogenetic analysis of a microbial community provides us with a benchmark for predicting the functional attributes of the studied microbiome (Gill et al., 2006), offering insights in how ecosystems respond to environmental changes.

The studies published to date have mainly focused on the characterization of community changes and the interactions within the bacteria kingdom, providing little information on the relationships among other co-existing microorganisms (e.g., fungi and archaea) and the environmental factors. Marine coastal sediment ecosystems are highly heterogeneous with high biodiversity and affected by spatial-temporal variations in environmental conditions (Cravo-Laureau and Duran, 2014). Low seawater temperature and low light intensity limit the removal of the oil in the Baltic sea in autumn and winter (Witt, 1995). The basin experiment presented here allows us to control the spatiotemporal effects and to focus on the successional changes in bacterial community in response to oil contamination. Hence, the basin experiment can offer an opportunity to focus particularly on the oil-microbe-environmental interactions in the well-defined experiment simulating conditions of the Baltic coastal area. The purpose was to investigate how oil degrading microbial communities evolve after an oil accident and what kind of species will be enriched in the summer season. The aims of the oil-contamination simulation experiment were (i) to study the oil distribution between surface water and the connected littoral sediment and bioremediation potential of oil contamination in a short period (8 weeks), (ii) to assess the successional changes in bacterial, fungal and archaeal abundance, community composition, and diversity in response to diesel oil contamination, and (iii) to investigate the speed of enrichment and identity, and origin (water or sediment) of oil-degrading microbes after an oil accident in the littoral sediment and the coastal surface water. We hypothesized that the microbial communities respond to diesel oil contamination over successional time. The ultimate goal is to shed light on the ecological impact of oil leaking accidents at the coast of the cold weather regions.

## Materials and Methods

### Experiment Setup

The 8-week basin experiment was established at the AlmaLab facilities of the University of Helsinki, in Lahti, Finland to simulate an diesel oil accident in a brackish Baltic Sea coast area. The seawater used in this experiment was taken from an uncontaminated area near Svartbäck in the Porvoo archipelago in the Gulf of Finland (60°27′33.0″N 25°50′96.0″E). Surface seawater was pumped into a fire truck and was transported to the lab immediately for the establishment of the experiment. Natural sand was taken from the shore of Korssundet, Porvoo, Finland (60°25′49.0″N 25°86′22.0″E) and was manually homogenized prior to the establishment of the experiment. To our knowledge, no significant oil spills occurred at these two sites in recent years. The physicochemical properties of the seawater and quarts sand used in this study are displayed in Table 1.

**TABLE 1:** Physicochemical properties of the seawater and quartz sand materials used in the study.

Property	Water	Natural sand	Artificial sand
pH	7.60	5.75	7.00
Cond. S	8.94	61	63
Oil g L^–1^	<LOQ	0.17	0.14
Total N	315	–	–
Salinity ppt	4.98	–	–
TOC ppm	10.28	–	–
DM	–	81.36%	75.75%
TOM	–	1.95%	0.76%

**FIGURE 1:**
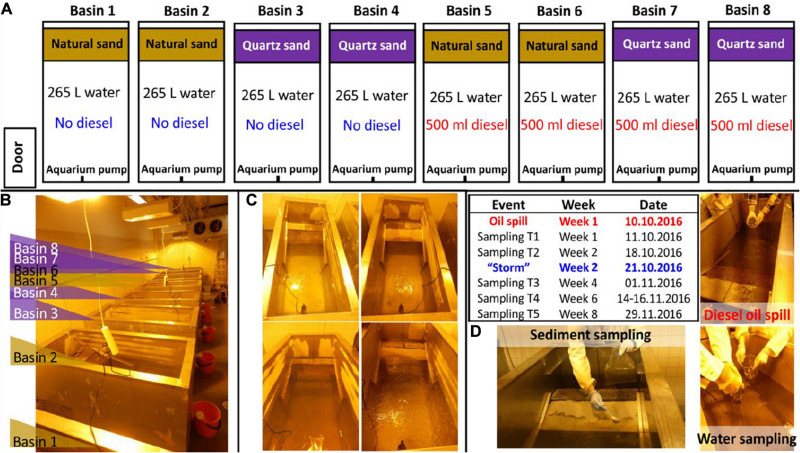
Oil simulation experiment framework: **(A)** experiment conceptual design, **(B)** image of the experiment setup, **(C)** images of the representative basins treated with natural sand without (up-left) and with oil contamination (down-left) and treated with quartz sand without (up-right) and with oil contamination (down-right) taken on the same day of the diesel oil accident and **(D)** graphic description of the diesel oil accident, the special event (“storm”), water and sediment sampling in the basins in a timeline.

The experiment (Figure 1) was set up in eight metal basins built with a shoreline (Suni et al., 2007). We thoroughly cleaned and disinfected the basins by 70% ethanol before use. Each basin was filled with 265 L of seawater (water depth 55 cm with 0.48 m^2^ surface area) and a littoral sediment area built of 18 kg of quartz sand or of 18 kg of natural littoral sediment (sediment depth about 8 cm). To simulate sea water movement, we installed an aquarium pump near the water surface in each basin. Basins 1–4 were designated as control basins without oil. Basins 5–8 were designated as diesel oil contaminated basins. The littoral zone represents the area submerged or exposed depending on water level (Acosta-González and Marqués, 2016). The Baltic Sea does not have tides, but the water level fluctuates (e.g., according to weather). Therefore, we used the term “littoral sediment” to describe the water-fluctuating sediment zone. Natural sand was used as the littoral sediment material at the shoreline in Basins 1, 2, 5, and 6, whereas autoclaved (120°C, 20 min) clean quartz sand was used as the littoral sediment in Basins 3, 4, 7, and 8. The diesel oil addition and sediment types resulted in two replications per each treatment. This experimental design can be statistically reinforced by including more basins. However, our air-conditioned laboratory space can accommodate maximum eight basins. Despite the compromises on the number of basin replications which might decrease the statistical power, this experimental design was still promising to assess the impact of diesel oil on microbial communities in the water and littoral sediment. A day-night cycle was created with lights on for 16 h and lights off for 8 h. Air temperature was set to 16°C and water temperature to 10°C. For Basins 5–8, 500 ml of diesel oil (NESTE Futura Diesel, summer quality, density at 0.84 g/ml) was spilled gently onto the surface of the water in each basin on October 10, 2016. On Week 2, stormy conditions were manually introduced to mimic the real natural storm in the unfrozen period in the coast of the Baltic Sea area. During this “storm,” oxygen and surface interruption was introduced by bubbling air through the water (20 L min^–1^ for 10 min) using an air pump (Millopore vacuum pump). The air hose was inserted to ca 30 cm below the surface and the bubbles efficiently disrupted the oil layer and the water surface.

### Sampling

The water and sediment were sampled five times in Week 1, 2, 4, 6, and 8 (Figure 1D). The first sampling was done one day after the diesel oil spill. At each sampling time, three surface water samples (c.a. 500 ml per sample, representing 0.19% of the tank volume) were gently taken from each basin to autoclaved glass bottles using a plastic tube water sampler (inner diameter: 5.3 cm inserted 23 cm into the water). One of the three water samples per basin was sent immediately to Alma Lab (Lahti, Finland) for diesel oil analysis, with the assumption that it represented a 22 cm^2^ area (= 0.35% share) of totally 6272 cm^2^ water surface in the tank. The seawater was re-filled to the basins after each sampling to maintain a stable water volume (265 L), but the diesel oil removed by sampling was not replaced. The share of diesel oil that was removed at each sampling (3 × 0.35% = ca 1%) was instead taken into account in the calculations of remaining diesel oil. Prokaryotic cells of the rest two water samples per basin were immediately collected using polycarbonate membrane filters (pore size 0.2 μm, diameter 47 mm; Whatman^TM^) prior to DNA extraction. For each sediment sample, four subsamples were taken at different positions along the shoreline. After being manually homogenized, c.a. 50 g of sediment materials per basin were pooled into a sterilized plastic sampling bag and sent to Alma Lab (Lahti, Finland) for diesel oil analysis. For molecular analysis, two sediment samples (15 g per sample) per basin were taken into sterile 50 mL falcon tubes and kept at −20°C freezer until being analyzed.

### Chemical Analysis

For diesel oil analysis, the non-volatile fraction of mineral oil (C10–C40) from petroleum products was extracted from sediment and water samples based on the standard procedure ISO 11046:2004, as modified by Jørgensen et al. (2005) and quantified according to ISO 16703:2004. Diesel oil analysis was done for all oil-contaminated samples. The diesel oil concentration in the control samples was determined only at Week 1 and Week 8 to ensure that these control samples were not cross-contaminated from basins in the same room. The basic chemical properties of the water (e.g., pH, salinity, electrical conductivity, total nitrogen and total organic carbon TOC) and sediment (e.g., pH, conductivity, dry matter and total organic matter TOM) samples taken from each control basin in Week 1 were measured by Eurofins Environment Testing Finland Oy (Lahti, Finland), based on standard procedures.

### DNA Extraction and PCR Amplification for Sequencing

Genomic DNA was extracted from c.a. 500 mg of homogenized sediment samples and from the membrane-filtered water samples using a PowerSoil DNA Isolation kit (MOBIO laboratories, Inc.) and quantified using the PicoGreen DNA assay (Invitrogen) following the manufacturers’ instructions. Bacterial partial bacterial and archaeal 16S rRNA gene and fungal internal transcribed spacer (ITS) regions were amplified for sequencing. The corresponding PCR primers, programs and reagents used are shown in Supplementary Table S1. The quality of the extracted DNA and PCR products were checked with agarose gel (1.5%) electrophoresis. A library was constructed and all sequences were generated with Illumina’s MiSeq platform using paired end reads at the Institute of Biotechnology, University of Helsinki, Finland.

### NGS Sequencing Analysis

The sequence data of both partial bacterial 16S rRNA gene and fungal ITS amplicon were analyzed using mothur V.1.39.5 (Schloss et al., 2009). The bacterial 16S rRNA sequence analysis followed the MiSeq SOP (Kozich et al., 2013). In fungal ITS sequence analysis, to permit pairwise alignment of fungal ITS sequences to calculate a pairwise distance matrix, we removed forward primer sequence (ITS1F), omitted all fungal ITS sequences that were less than 200 bp in length, and truncated the remaining sequences to the first 200 bp (Hui et al., 2017). For quality control, the sequences that contained ambiguous (N) bases and homopolymers longer than eight nucleotides were screened out. The remaining sequences were pre-clustered to allow for up to 2 bp difference to remove potential sequencing errors before the identification of the chimeric sequences using the UCHIME algorithm (Edgar et al., 2011). After removing the chimeric sequences, the unique sequences were classified with mothur-formatted UNITE taxonomy reference database (v. 7.2, released on 01.12.2017), using the default bootstrapping algorithm (cutoff value: 80%). Only fungal sequences were kept and assigned into OTUs by classification using fasta as the split method, based on nearest neighbor clustering at 97% similarity. Singletons were removed in both bacterial and fungal ITS datasets. The raw sequencing data were deposited in the NCBI Sequence Read Archive (SRA), BioProject ID PRJNA419286, accession no. SRP125392.

### Quantitative PCR (qPCR) Assays

The qPCR assay standards were a series of solution dilutions of *Cupriavidus necator* pJP4 (DSM 4058, complete genome length: 7,255,290 bp) for bacteria, *Saccharomyces cerevisiae* kanta 10 HAMBI (complete genome length: 12,100,000 bp) for fungi, *Sulfolobus acidocaldarius* (DSM 639, complete genome length: 2,225,959 bp) for archaea. The target gene copies per genome was checked via the ribosomal RNA operon copy number database (rrn DB) (Stoddard et al., 2015). The target gene copy numbers of the stock DNA solution were checked with the online DNA Copy Number and Dilution Calculator (ThermoFisher) with the known complete genome length, measured stock DNA concentration and the target gene copies per genome. The total bacterial 16S rRNA genes, fungal ITS genes and archaeal 16S rRNA genes were quantified using a LightCycler 96 qPCR machine (Roche Life Science). The corresponding qPCR primers, programs and reagents used are shown in Supplementary Table S2. All samples were run in triplicate.

In this study, we determined two alkane degrading genes, Cytochrome P450 (van Beilen et al., 2006) and *alkB* (Powell et al., 2006), and one PAH-ring hydroxylating dioxygenase gene from Gram negative bacteria PAH-RHD_α_ GN (Cébron et al., 2008) using DNA cloning method. For standards, PCR products of those above-mentioned genes were amplified from environmental samples with the primer sets listed in Supplementary Table S3, purified following the manufacturers’ instructions, cloned and transformed into competent *E. coli* cells according to the instructions of the Zero Blunt TOPO PCR Cloning Kit for Sequencing, with One Shot TOP10 Chemically Competent *E. coli* (Invitrogen, Finland). To ensure the success of cloning, the PCR products of bacterial 16S rRNA gene of these samples were sequenced at University of Helsinki Institute of Biotechnology (Viikki, Helsinki) and compared to the known sequences within U.S. NCBI Nucleotide collection (nr/nt) through Standard Nucleotide BLAST. The plasmids were purified following the instructions in GeneJET Plasmid Miniprep Kit (Thermo Scientific, Finland), ran through agarose gel electrophoresis and extracted from gel using the instructions on GeneJET Gel Extraction Kit (Thermo Scientific, Finland). Quant-iT PicoGreen dsDNA Assay Kit (Invitrogen, Finland) and Victor (Perkin Elmer) were used to quantify the total DNA concentration of the purified plasmid DNA according to the manufacturers’ instructions. The gene copy numbers of the standards were calculated using the standard curve, the weight of the plasmid and Thermo Fisher Scientific DNA Copy Number Calculator. The plasmid DNA was stored at −20°C and used to create standards through 10-fold dilution series. Quantitative PCR assays were carried out using a LightCycler 96 qPCR machine (Roche Life Science) with the corresponding qPCR programs and reagents as shown in Supplementary Table S3. All samples were run in triplicate.

### Statistical Analysis

The alpha diversity of bacterial and fungal communities of each sample was estimated at the sequencing depths of 1470 and 1107 reads per sample, respectively, using the R package *phyloseq* (McMurdie and Holmes, 2013). As diesel oil concentration, alpha diversity and the qPCR data were not normally distributed, non-parametric Wilcoxon test or Kruskal–Wallis rank sum test was used to test whether the means differed between two or over two treatment groups, respectively. Tukey multiple comparisons of means were used when the differences were significant between groups. Significance of the differences was concluded at 95% confidence level (alpha < 0.05).

The abundance of each OTU was normalized against the sum of the total OTU numbers in each sample. Principal coordinate analysis (PCoA) was applied to the normalized data to visualize broad pattern of bacterial and fungal community data based on weighted UniFrac distance, using the R package *phyloseq* (McMurdie and Holmes, 2013). PERMANOVA was used to assess whether the treatments and their interaction resulted in a different community composition with the default 9,999 permutations. Betadisper was used to test whether the dispersions of observations between the treatment groups were equal with 9,999 permutations. PERMANOVA and Betadisper were performed based on weighted UniFrac distance using the R package *Vegan* (Oksanen et al., 2007). Significance of the differences was concluded at 95% confidence level (alpha < 0.05).

The raw microbial OTU count data were used to explore the oil-responding OTUs that showed significantly differential abundance between oil-contaminated and control basins with R package *DESeq2* (Love et al., 2014). False discovery rate (FDR) method was used to adjust the *p*-values. The rare OTUs (relative abundance < 0.05%) were removed prior to DESeq2 analysis. The OTUs that had Log2FoldChange of relative abundance over 3 and adjusted *p*-values less than 0.001 were identified as oil-responding OTUs and displayed in bar charts using R package *ggplot2* (Wickham, 2016).

## Results

### Diesel Oil Concentration

**FIGURE 2:**
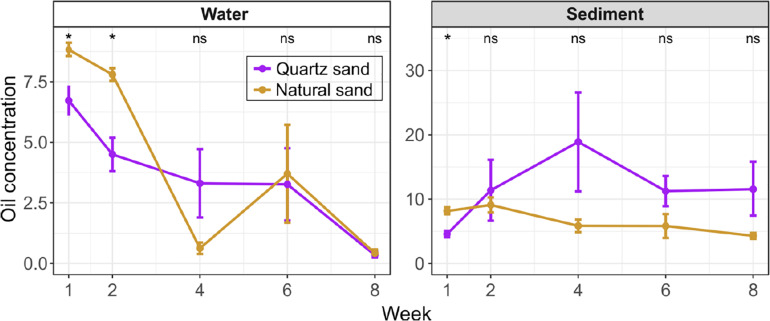
Oil concentration in the surface water (unit: g L-1) and the littoral sediment (unit: g kg-1) of the oil-contaminated basins over time. Error bars represent standard error of means. The effect of sand type was tested with Wilcoxon test at each sampling time: “*” *p* < 0.05 and “ns” not significant. In water, the oil concentration was below the detection limit of instrument in the non-contaminated control basins at Week 1. In the sediment, the oil concentration in the non-contaminated control basins was low (0.1 g kg-1 in quartz sand and 0.2 g kg-1 in natural sand at Week 1 and undetected at Week 8).

In the oil-contaminated water, oil concentration notably decreased over time (Figure 2). The initial oil concentration was 6.7 g L^–1^ and 8.8 g L^–1^ in the surface water connected with quartz sand and natural sediment, respectively (Supplementary Figure S2). Until Week 8, 95.09% of diesel oil was removed from water on average. Based on share of surface area, each sampling removed 1% of the diesel oil floating on the surface. Thus, the total amount of diesel oil removed by sampling is below 5% even if there was no degradation. Compared to the actual removal of diesel oil seen, the sampling share is negligible.

In the initial 2 weeks, the water oil concentration was greater in basins with natural sand than with quartz sand. In both types of basins, the water oil concentration significantly decreased (Wilcoxon test, *p* < 0.05) by Week 2 (8 days after diesel oil spills). After Week 2, we observed oil films still covering water surfaces of all contaminated basins, despite the slow water agitation by the pump. In order to improve simulation of field conditions and thereby the oxygen availability in the water (Figure 1), we conducted simulated a storm using an air pump. At Week 4, a floating turbid mass appeared on the surface of basin 5 and 6, with natural sand and diesel oil. This mass may also have resulted in unequal distribution of the oil in the basins. At Week 4 and Week 6 the water oil concentration in the quartz sand basin remained steady (3.3 g L^–1^), while the diesel oil concentration fluctuated in natural sand basins, starting from 0.6 g L^–1^ at Week 4 followed by a sharp increase 3.7 g L^–1^ at Week 6 (Supplementary Figure S2). The water oil concentration in basins with both quartz and natural sand reached the similar level (0.3 ∼ 0.4 g L^–1^) in 8 weeks.

As assumed, the natural sand had a higher organic matter contents than the quartz sand (Supplementary Figure S3). Except for Week 1, the diesel oil concentration was generally lower in the natural sand than the quartz sand throughout the experiment (Figure 2). In the natural sand sediment, the diesel oil level slowly decreased from a maximum of 9.1 g kg^–1^ at Week 2 down to 4.3 g kg^–1^ at Week 8. In contrast, the diesel oil level in the quartz sand rose rapidly and peaked at 18.9 g kg^–1^ at Week 4, after which degradation decreased the level to 11.6 g kg^–1^. The lower maximum in the natural sand may reflect the presence here of oil-degrading microbes.

In the control basins, diesel oil concentration in the surface water was below the detection limit of instrument at the beginning and end of the experiment. The recorded diesel oil concentration in the littoral sediment in the control basins was low (0.1 g kg^–1^ in quartz sand and 0.2 g kg^–1^ in natural sand) at the beginning and below detection at the end of the experiment. This background apparently represents organic matter in the sediment as was also seen before (Yan et al., 2018b).

### Shared Microbial Taxa and Taxonomic Composition

For comparison of the microbial community composition between the four compartments quartz sand, water connected to quartz sand, natural sand, and water connected to natural sand, the sequencing data of different time points were pooled.

**FIGURE 3:**
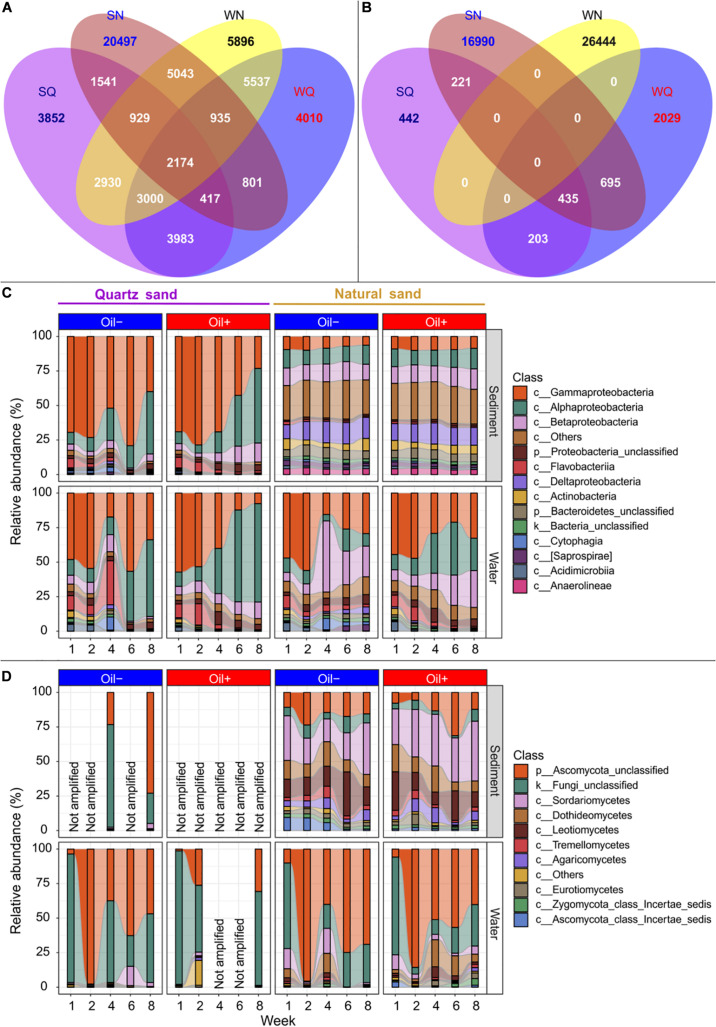
Microbial community composition. Venn diagram showing the number of the detected bacterial **(A)** and fungal **(B)** OTUs that were shared between quartz sediment (SA), natural sediment (SN), surface water connected with quartz sand (WA) and surface water connected with natural sand (WN) regardless of oil contamination, using R package VennDiagram (Chen and Boutros, 2011). Bacterial **(C)** and fungal **(D)** community composition at the class level between different treatments over time. The rare classes (average relative abundance < 1% in the whole dataset) were grouped into “c__Others” in the figure. Fungal partial ITS genes could not be amplified (expressed as “Not amplified” in the figure) in the oil-contaminated basins at some sampling time points or in the sterilized quartz sediment.

We detected a total of 18,826 bacterial OTUs in the quartz sand sediment, 32,337 in the natural sediment, 20,857 in water connected with quartz sand and 26,444 in water connected with natural sediment (Figure 3A). The four environmental types shared 2,174 bacterial OTUs. There were 9,574 bacterial OTUs shared between the quartz sand and the connected surface water, accounting for 55.4% of the total number of unique bacterial OTUs in the quartz sand. The natural sediment shared 9,081 bacterial OTUs with the surface water connected with natural sediment (28.1%).

We observed a total of 1,301 fungal OTUs in the quartz sand sediment, 18,341 in the natural sediment, 3,362 in the water connected with quartz sand and 7,141 in the water connected with the natural sediment (Figure 3B). Unlike bacteria, no common fungal OTUs were shared among all four environmental types. The quartz sand shared 638 (59.1%) of its unique fungal OTUs with the connected surface water. The natural sediment shared 2 856 fungal OTUs with the surface water connected with natural sediment (15.6%).

The bacterial community composition was summarized based on sand type, diesel oil contamination and time (Figure 3C). In water connected to sterilized quartz sand, Gammaproteobacteria (on average 38.05%) and Alphaproteobacteria (31.71%) were the major classes. In water connected with natural sand, Gammaproteobacteria (34.82%), Alphaproteobacteria (16.34%), Betaproteobacteria (18.86%) were predominant. Other bacterial classes such as Epsilonproteobacteria (1.58%) and Verrucomicrobiae (1.31%) accounted for 1.93 and 9.27% of the bacterial community in water connected to the quartz and natural sediment, respectively. Bacterial community in the quartz sand were similar to that in the connected surface water, dominated by Gammaprotebacteria (59.59%) and Alphaproteobacteria (22.32%). In natural sediment, Proteobacteria such as Deltaproteobacteria (13.63%), Betaproteobacteria (12.69%), Alphaproteobacteria (12.23%), and Gammaproteobacteria (8.84%) accounted for the majority in the bacterial community. In addition, other bacterial classes, such as Bacteriodia (2.85%), Pedosphaerae (1.27%), Clostridia (1.25%), Spirochaetes (1.29%), and Solibacteres (1.12%), accounted for 26.37% of the whole bacterial community in natural sediment. There was an apparent shift of relative abundance of bacterial taxa in the quartz sand and the connected water in the oil-contaminated basins over time, compared with the control basins. For example, Gammaproteobacteria decreased, whereas Alphaproteobacteria increased in relative abundance with time in the presence of oil contamination. In the natural sediment, the effect of time and oil contamination on bacterial community composition was not as evident as in water. In water connected with natural sand, the relative abundance of Alphaproteobacteria increased with time in the presence of diesel oil.

In water connected with quartz sand in the control basins, the majority of the detected fungal OTUs belonged to unclassified Fungi (44.96%) and unclassified *Ascomycota* (49.63%) (Figure 3D). In water connected with quartz sand in the oil-contaminated basins, fungal ITS genes were only amplified at Week 1, 2, and 8 and the majority (90.39%) of the detected OTUs were the unclassified groups. Other classes (in total 6.43%), such as Chytridiomycetes (3.01%), Saccharomycetes (1.99%), and Blastocladiomycetes (1.29%), were evidently more abundant in the oil-contaminated water at Week 2. In water connected with natural sediment, the unclassified groups accounted for 84.40 and 74.84% of the fungal community in the uncontaminated and oil-contaminated basins, respectively. Natural sand increased the abundance of Dothideomycetes, Sordaromycetes, and Leotiomycetes in water compared to quartz sand, regardless of diesel oil contamination. In quartz sand sediment, fungal ITS could hardly be detected. The difference in fungal community composition at class level between the uncontaminated and oil-contaminated natural sediment was obvious. In contrast to the water community, the unclassified fungi only accounted for 6.22% in the natural sediment community. The natural uncontaminated sediment harbored a community of 29.71% Sordariomycetes, 16.83% Leotiomycetes, 15.18% unclassified Ascomycota, 12.28% Dothideomycetes, 5.83% Agaricomycetes, 3.71% Tremellomycetes, 3.38% Ascomycota class Incertae sedis, 2.86% Eurotiomycetes, and 2.23% Zygomycota class Incertae sedis.

We did not successfully amplify archaeal 16S rRNA gene in all samples. The taxonomic composition of the archaeal community in the basins based on the incomplete data is shown in Supplementary Figure S1. The majority of the archaeal sequences were unclassified. Methanomicrobia, Parvachaea, DSEG, Thaumarchaeota, Thermoprotei, unclassified Crenarchaeota, and unclassified Euryarchaeota were found in both the sediment and the surface water of the basins.

### Microbial Diversity and Abundance

Shannon diversity values were computed as an estimation of the microbial alpha diversity in the sediment and water samples. The mean bacterial Shannon diversity was 5.32 in quartz sand sediment, 6.82 in natural sediment, 5.57 in water connected with quartz sand sediment, and 5.86 in water connected with natural sediment on average (Supplementary Figure S4a). Bacterial Shannon diversity was greater in both sediment and water in basins with natural sand than with quartz sand (*p* < 0.05, Table 2). Particularly at Week 4, 6, and 8, diesel oil contamination significantly increased bacterial diversity in water with quartz sand sediment (*p* < 0.05, Supplementary Figure S4a). In the natural sediment, bacterial diversity was not affected by diesel oil contamination (Supplementary Figure S4a).

**TABLE 2:** Non-parametric testing of bacterial and fungal alpha diversity index (Shannon) in water and sediment.

Kingdom	Ecosystem	Non-parametric testing			
			df	Chi-squared	Significance
Bacteria	Water + Sediment	Ecosystem	1	14.98	***
	Water	Time	4	33.57	***
		Oil	1	5.51	*
		Sand	1	6.90	**
		SandOil	3	13.68	**
	Sediment	Time	4	3.12	ns
		Oil	1	1.00	ns
		Sand	1	59.26	***
		SandOil	3	61.87	***
Fungi	Water + Sediment	Ecosystem	1	29.15	***
	Water	Time	4	19.97	**
		Oil	1	0.71	ns
		Sand	1	3.41	ns
		SandOil	3	49.75	***
	Sediment	Time	4	9.63	*
		Oil	1	0.19	ns
		Sand	1	14.79	***
		SandOil	3	15.99	***

The effect of oil contamination (oil), ecosystem type (ecosystem, water vs. sediment) and sand type (sand, natural sand vs. artificial sand) was evaluated using one-way Wilcoxon test, respectively. The effect of sampling time (time) and the interaction term of sand type and oil contamination (SandOil) was tested with one-way Kruskal–Wallis test. *** p < 0.001, ** p < 0.01, * p < 0.05 and “ns” not significant.

Fungal Shannon diversity was 1.96 in quartz sand sediment, 3.65 in natural sediment, 1.99 in water connected with quartz sand sediment and 2.44 in water connected with natural sediment on average (Supplementary Figure S4b). Similar as bacteria, fungi was more diverse in natural sediment than in water (*p* < 0.05, Table 2). Diesel oil had no effect on fungal diversity, regardless of the ecosystems (Table 2).

DNA concentration was used as a proxy for total microbial biomass. The DNA concentration remained stable in the sediment, but changed over time in the water (Supplementary Figure S5). It was evident that the concentration of DNA extracted from sterilized quartz sand was low throughout the experiment. Natural sand generally increased the concentration of microbial DNA not only in the sediment itself, but also in the water throughout the experiment, compared to the sterilized quartz sand and the water in these basins. Diesel oil had no impact on microbial DNA concentration, regardless of the ecosystems.

The total gene copies of bacterial 16S rRNA, fungal ITS and archaeal 16S rRNA were quantified to reveal the bacterial, fungal, and archaeal abundance, respectively, in the basin experiment.

**FIGURE 4:**
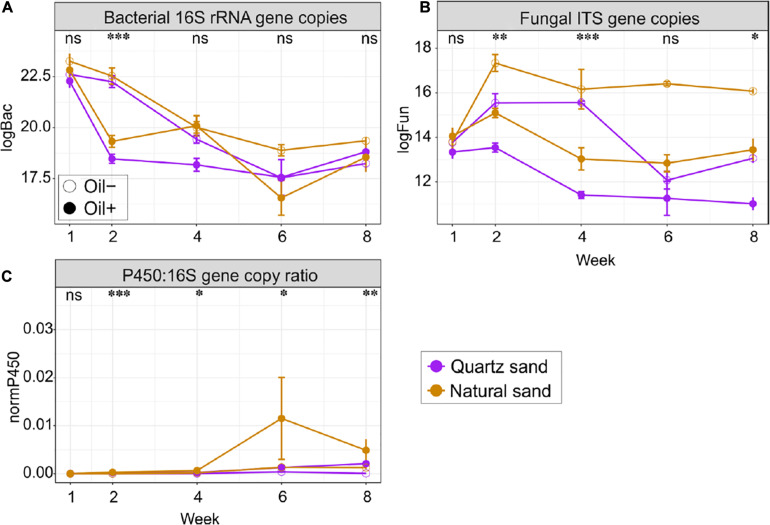
Quantitative analysis of **(A)** bacterial 16S copies, **(B)** fungal ITS copies, and **(C)** copy ratio of P450 gene to bacterial 16S rRNA gene in the surface water in the basins over time. Abbreviations and units: logBac natural logarithm transformed values of bacterial 16S rRNA gene copy; logFun natural logarithm transformed values of fungal ITS copies; P450:16S gene copy ratio absolute copies ratio between P450 and bacterial 16S rRNA gene; All marker genes were measured in copies per gram of oven dry sand or per litter of water, depending on the ecosystems. The effect of oil contamination on the gene copies or the copy ratio was tested with Wilcoxon test: ^∗∗∗^*p* < 0.001, ^∗∗^*p* < 0.01, ^∗^*p* < 0.05 and “ns” not significant.

Bacterial abundance did not change over time, regardless of the ecosystem (Figure 4A and Supplementary Figures S2, S3). In the sediment, diesel oil contamination did not influence bacterial abundance. In water, however, diesel oil contamination significantly decreased bacterial abundance, especially at Week 2. After Week 2, we did not observe any difference in bacterial abundance in water between oil-contaminated and control basins. In the sediment, bacterial abundance was significantly higher in natural sand than in the quartz sand, but the effect of sand type on bacterial abundance was not evident in water.

Fungal abundance changed with time in water, but not in sediment (Figure 4B and Supplementary Figures S2, S3). Diesel oil significantly decreased fungal abundance in both sediment and water. The negative effect of diesel oil contamination on fungal abundance in water was remarkable at Week 2, 4, and 8. Natural sand increased fungal abundance in both sediment and water.

The qPCR analysis using the archaeal primers contained a high proportion of bacterial 16S rRNA gene sequences (data not shown) and the archaeal qPCR results are therefore not presented in the manuscript. The copy ratio of P450 gene to bacterial 16S rRNA gene were used to define the proportion of the oil-degrading populations in the total bacterial community (Figure 4C and Supplementary Figures S2, S3). Initially in the control basins, the copy ratio, was 2.19% in quartz sand sediment, 9.37% in natural sediment, 0.09% in water connected with quartz sand sediment and 0.07% in water connected with natural sediment. Generally, the ratio increased over time in both ecosystems. Diesel oil contamination had no impact on the copy ratio in sediment, but significantly increased the copy ratio in water. In water, the positive effect of diesel oil contamination on the copy ratio started at Week 2 and lasted until the end of the experiment (Figure 4C). Overall, the proportion of oil-degrading bacteria (P450:16S rRNA gene copy ratio) was 7.16 times higher in water connected with quartz sand and 6.14 times higher in water connected with natural sand in oil-contaminated basins than in the control samples. However, sand type did not affect the copy ratio in water (Supplementary Figures S2, S3).

The qPCR amplification of *alkB* and PAH-RHD GN genes in our samples were only partly successful and not reliable (Supplementary Figures S2, S3).

### Community Level Response

**FIGURE 5:**
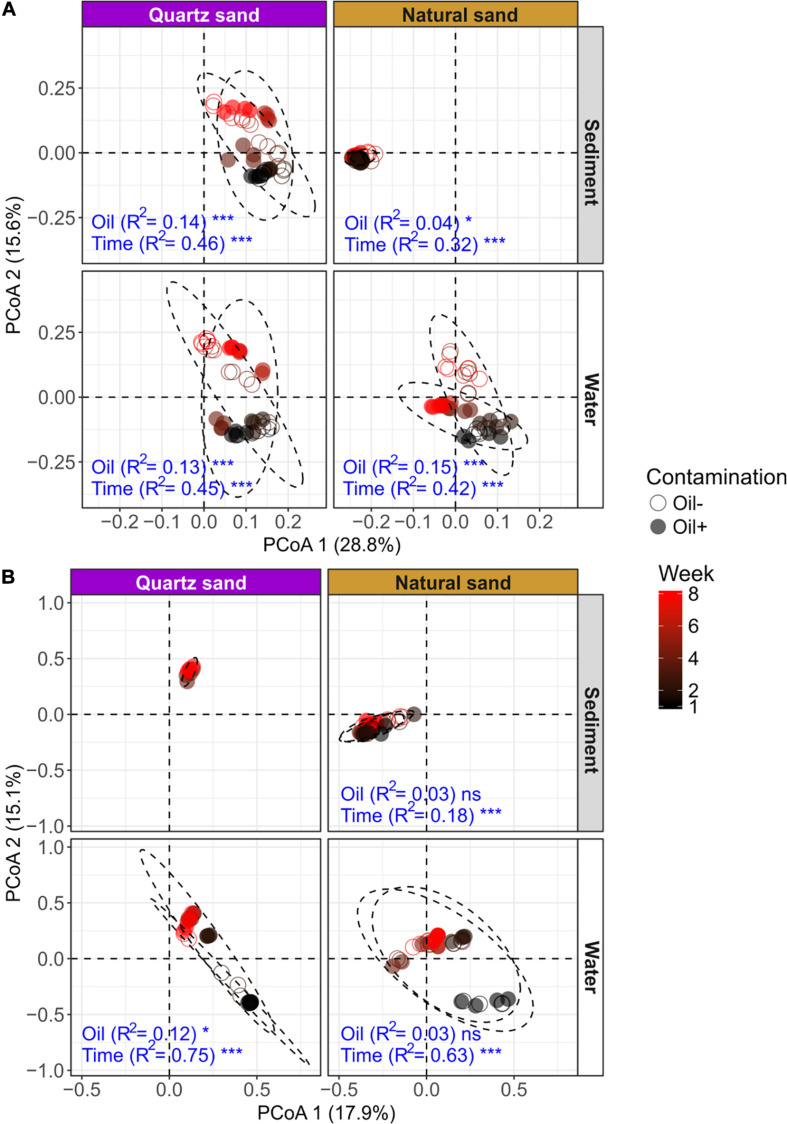
Unconstrained ordinations (PCoA) on microbial community distribution based on weighted UniFrac distance: **(A)** bacterial community and **(B)** fungal community. The effects of oil contamination (oil) and sampling time (time) on bacterial and fungal communities in different ecosystems (sediment and water) and sand types (quartz and natural) were tested using PERMANOVA based on weighted UniFrac distance, respectively. The R2 and *p*-values (shown as significance) exported from PERMANOVA tests (factors: oil contamination and time) were shown in blue texts. The significance was tested with 9,999 permutations: ***p < 0.001, *p < 0.05, and “ns” not significant. The effect of oil contamination and time on fungal community in the autoclaved quartz sand was not tested, due to a failure in PCR amplification of the fungal ITS sequences.

PERMANOVA results showed that the bacterial community composition was distinct between sediment and water (Table 3). Time was the overriding factor which solely explained high variations in bacterial communities in the surface water (32%) and in the littoral sediment (11%). The successional changes in bacterial community composition over time (expressed as sampling “Week”) was visualized in the unconstrained ordinations (PCoA) (Figure 5A). Diesel oil contamination significantly altered bacterial community composition, solely explaining 10% of the total variations in the surface water and 3% in the littoral sediment. Sand type had significant effect on the bacterial community composition in both the littoral sediment and the connected surface water. Bacterial community composition differed between natural and quartz sand in sediment (*p* < 0.001, *R*^2^ = 0.30) and in water (*p* < 0.001, *R*^2^ = 0.05, Table 3). Bacterial community structure in the sterilized quartz sand sediment showed high similarity and similar successional pattern as that in the surface water (Figure 5A). Natural sediment influenced the bacterial community in the connected surface water. Nevertheless, bacterial communities in the natural sediment showed lower dissimilarity in composition as revealed by lower variations in the space of PCoA coordination, compared to those in the surface water.

**TABLE 3:** PEMANOVA table for the analysis of the weighted UniFrac distance matrix to test the main effects of ecosystem (ecosystem), sand type (sand), oil contamination (oil), and their interaction terms on bacterial and fungal community.

Kingdom	Ecosystem	PERMANOVA				Betadisper
			df	*R*^2^	Pr (>F)	Pr (>F)
Bacteria	Water + Sediment	Ecosystem	1	0.0702	***	ns
	Water	Time	4	0.3195	***	***
		Oil	1	0.0962	***	**
		Sand	1	0.0698	***	ns
		SandOil	3	0.1981	***	**
		OilTime	9	0.5455	***	***
		SandTime	9	0.4776	***	***
	Sediment	Time	4	0.1137	***	ns
		Oil	1	0.0331	*	ns
		Sand	1	0.5148	***	***
		SandOil	3	0.5742	***	***
		OilTime	9	0.1975	**	**
		SandTime	9	0.7262	***	***
Fungi	Water + Sediment	Ecosystem	1	0.1232	***	***
	Water	Time	4	0.5038	***	***
		Oil	1	0.0343	*	*
		Sand	1	0.0773	***	ns
		SandOil	3	0.1388	**	**
		OilTime	9	0.6122	***	***
		SandTime	9	0.7030	***	***
	Sediment	Time	4	0.1827	***	*
		Oil	1	0.0482	*	ns
		Sand	1	0.2555	***	*
		SandOil	3	0.2758	***	*
		OilTime	9	0.3073	***	ns
		SandTime	9	0.4349	***	**

The effect of oil contamination (oil), ecosystem type (ecosystem, water vs. sediment) and sand type (sand, natural sand vs. artificial sand) was evaluated using one-way Wilcoxon test, respectively. The effect of sampling time (time), the interaction terms of sand type and oil contamination (SandOil), oil contamination and sampling time (OilTime), and sand type and sampling time (SandTime) were tested with one-way Kruskal–Wallis test. *** p < 0.001, ** p < 0.01, * p < 0.05 and “ns” not significant.

Fungal community composition significantly differed between natural and quartz sand in sediment (*p* < 0.001, *R*^2^ = 0.20) and in water (*p* < 0.001, *R*^2^ = 0.08, Table 3). The temporal effect was obvious, solely explaining large variations in fungal community composition in both littoral sediment (*p* < 0.001, *R*^2^ = 0.18) and surface water (*p* < 0.001, *R*^2^ = 0.50, Table 3). The fungal community in the natural sediment was not affected by diesel oil contamination. Due to the fact that fungal ITS was not amplified in the quartz sediment samples, the diesel oil effect on fungal community in quartz sediment was not tested with PERMANOVA. The effect of diesel oil contamination on the fungal community was significant in the surface water connected to sterilized quartz sand (*p* < 0.001, *R*^2^ = 0.12), but not significant in the surface water connected to natural sand (Figure 5B). As with the bacterial community distribution pattern, fungal communities in the sterilized quartz sand sediment showed high similarity (short distance) to those in water.

### Oil-Responding OTUs

Here oil-favored OTUs were defined as those with increased relative abundance, whereas oil-depressed ones were those with reduced relative abundance in the presence of diesel oil.

**FIGURE 6:**
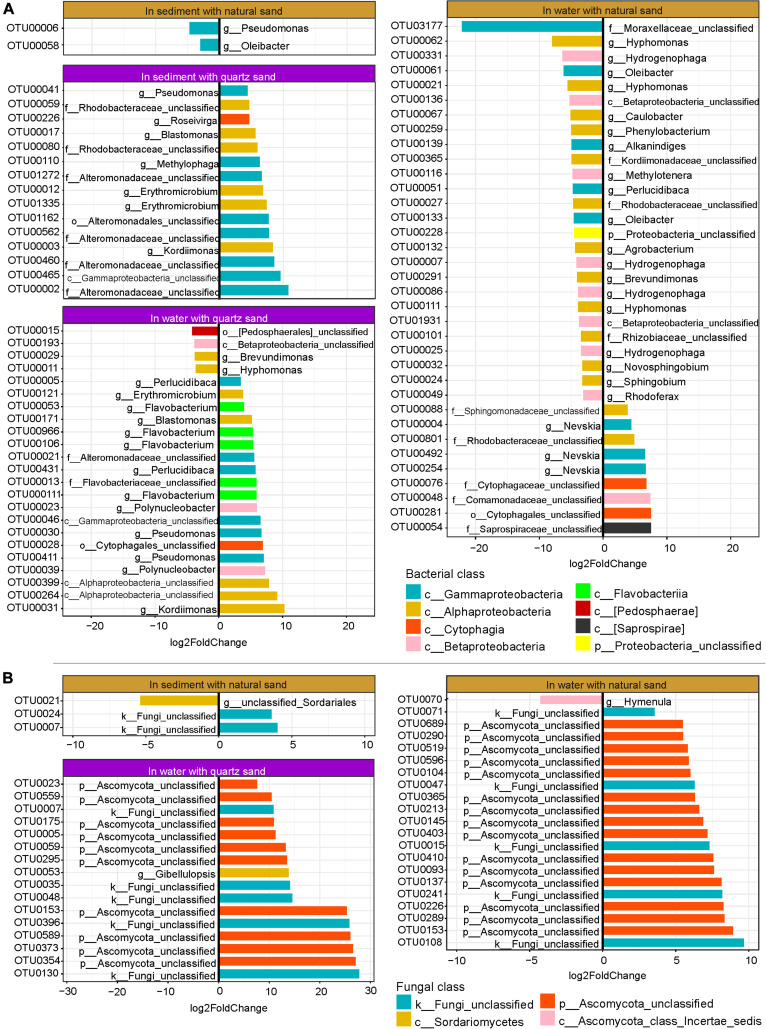
Oil-specific bacterial **(A)** and fungal OTUs **(B)** detected by DESeq2. The rare OTUs (relative abundance < 0.05%) were removed prior to DESeq2 analysis (Log2FoldChange > 3, *p* < 0.001).

In water connected with sterilized quartz sand, 23 bacterial OTUs showed abundance changes in response to diesel oil contamination in water, of which 19 bacterial OTUs were oil-favored (Figure 6A). Among the oil-favored OTUs, there were 5 Alphaproteobacteria (e.g., Erythromicrobium, Blastomonas, Kordiimonas, and unclassified Alphaproteobacteria), 2 Betaproteobacteria (Polynucleobacter), 6 Gammaproteobacteria (e.g., Perlucidibaca, Pseudomonas, unclassified Alteromonadaceae and unclassified Gammaproteobacteria), 5 Flavobacteriia (e.g., Flavobacterium and unclassified Flavobaceteriaceae) and one Cytophagia (unclassified Cytophagales).

In water connected with natural sand, 35 bacterial OTUs (relative abundance ≥ 0.05%) showed changes in relative abundance between the oil-contaminated and control samples, of which 9 were oil-favored (Figure 6A). Of the oil-favored OTUs, two belonged to Alphaproteobacteria (e.g., unclassified Sphingomonadaceae and Rhodobacteraceae), one Betaproteobacteria (unclassified Comamonadaceae), three Gammaproteobacteria (genus Nevskia) and two Cytophagia (unclassified Cytophagales).

In sediment with sterilized quartz sand, all of the 15 bacterial OTUs that significantly responded to diesel oil contamination were oil-favored (Figure 6A). Among the oil-favored OTUs, there were 6 Alphaproteobacteria (e.g., Erythromicrobium, Blastonomas, Kordiimonas, and unclassified Rhodobacteraceae), 8 Gammaproteobacteria (e.g., Pseudomonas, Methylophaga, unclassified Alteromonadaceae and unclassified Gammaproteobacteria), 5 Flavobacteriia (e.g., Flavobacterium and unclassified Flavobaceteriaceae) and one Cytophagia (unclassified Cytophagales).

In natural sand sediment, 2 bacterial OTUs had differential abundance between the oil-contaminated and control samples, both of which were oil-depressed (Figure 6A).

As the fungal ITS sequences were not properly amplified in the quartz sand sediment contaminated with diesel oil, DESeq2 was not performed. In water with quartz sand, 16 fungal OTUs changed significantly in relative abundance, all of which were oil-favored (Figure 6B). They include ten unclassified Ascomycota, five unclassified fungi and one Sordariomycetes (Gibellulopsis). In water connected with natural sand, 20 fungal OTUs responded to diesel oil contamination, among which the majority were oil-favored including 14 unclassified Ascomycota OTUs and 5 unclassified fungi. The only oil-depressed OTU belongs to Hymenula.

In sediment with natural sand, three fungal OTUs changed in relative abundance in the presence of diesel oil contamination, of which two unclassified fungi were oil-favored and one unclassified Sordariales was oil-depressed (Figure 6B).

## Discussion

### Distribution and Degradation of Diesel Oil

Diesel oil concentration was monitored in oil-contaminated basins in both the surface water and the littoral sediment over time. The decrease of diesel oil in water was evident. Harayama et al. (1999) summarized the behavior of petroleum in marine environments. Once diesel oil is spilled, it spreads over the surface of the water. Then the hydrocarbons are subjected to weathering through the physiochemical processes of evaporation of the low-molecular-weight factions, dissolution of the water-soluble components, mixing of the diesel oil droplets with seawater, photochemical oxidation and biodegradation. In our study, the lighter volatile fractions of hydrocarbons evaporated during the first few weeks. The smell of hydrocarbons was strong in the experiment room the first week. The medium- to high-molecular-weight fractions were potentially subjected to biodegradation, sampling removal and sorption into the sediment. The variation of diesel oil concentration in the replicated basins can be attributable to the heterogeneous distribution of diesel oil in the environment, especially in water. Over the 8-week experiment, only 5% of the diesel oil was left in water. However, these influencing conditions in our basin system did not permit a precise calculation on how diesel oil was balanced between water and sediment.

In the 8-week basin simulation experiment, diesel oil was detected with low but stable concentration in the littoral sediment in the oil-contaminated basins over time. One day after the diesel oil spill, diesel oil concentration detected in the sediment of the oil-contaminated basins were 40 times higher than the controls, regardless of the sand type, indicating rapid diffusion of diesel oil from water to the sediment in our system. The effect of sand type on diesel oil concentration in the surface water and the littoral sediment was only detected immediately after the diesel oil spill. Diesel oil is constituted with complex hydrocarbon fractions with varied degrees of hydrophobicity, and it can be expected that the accidental spill event could change the physico-chemical and biological properties of water as well as the nearby sediment. TOM was found constantly higher in natural sand than in quartz sand, likely owing to the sorption of diesel oil to the organic matter in the natural sand. In analogy with our observations, a linear relationship was detected between PAH concentration and total organic carbon (Witt, 1995). Soil and sediment contain various organic materials, among which humic substances, kerogen and black carbon are the dominant organic components that can largely affect the sorption and desorption of hydrophobic organic chemicals (Huang and Weber, 1997; Weber et al., 1998; Huang et al., 2003). This sorption process reduces the mobility, chemical and biological reactivity, and bioavailability of hydrophobic organic chemicals in surface aquatic and groundwater systems (Huang et al., 2003), which may lead to a decreased rate and extent of biodegradation (Trindade et al., 2005). The oil concentration in the sediment was stable over time, likely suggesting an equilibrium of sorption, desorption and biodegradation processes of hydrocarbons.

In this study, the artificial storm was introduced to mimic the natural coastal condition to allow strong mixing of water and air in the system in a short time. The storm itself interrupted the oil film on the water, mixed the water, and made the water under the oil more aerobic. We found that the sharp loss of diesel oil in the water samples following the storm was more evident in water connected to natural sand than in water connected to quartz sand. The reason could be that the natural sand microbes had a much better degradation potential, and that all they needed was some oxygen, while the quartz sand basins were not helped by the oxygen to the same degree. Alternatively, the diesel oil could have been moved into and sorbed faster into the SOM of the natural sand than the (SOM free) quartz sand and was quickly degraded by microbes in natural sand.

Because of the presumed unevenness of the diesel oil in the sediment (highest at the water-sand interphase) an average diesel oil concentration in the sediment was not calculated, and therefore a mass balance calculations of the total amount of oil in the basis system was not attempted. It can be concluded, however, that when the oil concentrations went down in both the sediment and in the water, the total oil amount was decreasing. Evaporation of diesel oil fractions was not monitored, but at a water temperature of 10°C and air temperature of 15°C the effect of evaporation was presumed not to play a major role in diesel oil removal. Furthermore, the main focus of this study was in following the influence of diesel oil on the microbial composition in the water and in the sediment.

### Taxonomic Composition of Microbial Communities

The bacterial communities in the surface water and the littoral sediment were dominated by Proteobacteria, e.g., Gammaprotebacteria, Alphaproteobacteria, and Betaproteobacteria, in line with other studies that reveal the ubiquitousness and dominance of Proteobacteria in marine environments (González and Moran, 1997; Acosta-González and Marqués, 2016). In the 8-week experiment, the quartz sand shared less than half of its bacterial and fungal OTUs with the connected surface water, revealing that a large number of microbes in the water did not reach the quartz sediment with the current setup. The result might also indicate other origins of microbes, e.g., from the air. The storm simulation may have caused aerosols that spread microbes between the basins.

The unclassified *Fungi* and unclassified *Ascomycota* accounted for the majority of the fungal ITS sequences retrieved from the water. The majority of these OTUs do not belong to any member of the described fungal taxa and are likely to be novel fungal lineages. Fungal OTUs detected in the sediment were more diverse and well classified with the latest updated UNITE database. Sordariomycetes, Leotiomycetes, unclassified Ascomycota, Dothideomycetes, Agaricomycetes, and Tremellomycetes dominated the fungal community in the natural sediment. The presence and ecological functions of these fungal lineages have been widely reported in marine environments (Kupper et al., 2006; Richards et al., 2012). No common fungal OTUs were shared among the four environmental types, reflecting the specificity of niche requirements for different fungi between water and the natural sand.

We found a large number of OTUs belonging to unclassified archaea in both sediment and water of the basins. The appearance of Methanomicrobia, Parvachaea, DSEG, Thaumarchaeota, Thermoprotei, unclassified Crenarchaeota, and unclassified Euryarchaeota in the samples agrees with our earlier report (Yan et al., 2018b) on archaeal community composition in the same region as used here. An observed difference was that Halobacteria, the most predominant class detected in natural environmental samples at the coast of the Gulf of Finland, were not detected in our basins. Taking into account that archaea were not amplified well, the basin experiment may have failed to detect some groups of archaea, especially Halobacteria.

### Changes in Microbial Diversity and Abundance

The compositions and structures of microbial communities are nowadays expressed as the richness and abundance of the molecular markers (such as rRNA genes, functional genes, and intergenic regions) with culture-independent approaches (Zhou et al., 2011). Diversity indices such as Shannon and Simpson are more robust for quantifying and comparing microbial diversity between samples than species richness estimators (Haegeman et al., 2013). Hence, in this study we used Shannon index to compare microbial taxonomic diversity between samples and the quantitative PCR amplification of selected marker genes to illustrate the abundance changes in bacteria, fungal, archaeal, and the selected oil-degrading communities.

Both sediment and water harbored high diversity of bacteria and fungi. Microbial biomass (estimated using DNA concentration), bacterial and fungal diversity were significantly higher in natural sediment than in water. This study highly agrees with the finding that coastal sediments harbor highest bacterial diversity within the marine realms in a global survey (Zinger et al., 2011) and that marine environments harbor highly diverse uncultured fungi, but less diverse than terrestrial environments (Richards et al., 2012). The diversity and abundance of planktonic microbes (e.g., bacteria and fungi) are driven by the primary production, availability of organic matter and nutrients, and redox gradient (Wang et al., 2012; Nemergut et al., 2013). Surface water environments low in nutrients are unlikely ideal for fungi that rely primarily on attachment to larger physical substrates and osmotrophy due to the loss of secreted enzymes and target nutrients by rapid diffusion in the open water (Richards et al., 2012). The natural sand markedly contributed to the high microbial diversity and OTU numbers detected in the surface water via the direct connection. This connection between the natural sediment and water made the seeding of each other’s diversity possible as reported earlier (Helmus et al., 2007; Shade, 2017).

In surface water, diesel oil contamination significantly increased bacterial diversity but decrease bacterial 16S rRNA gene abundance. The increase in microbial diversity is hypothesized as the result of the creation of niches as disturbances cause environmental heterogeneity (Galand et al., 2016). The decrease of bacterial abundance might indicate the negative impact of diesel oil contamination on bacteria in the water. As oil was detected in low concentration in the natural sediment, it was not surprising that the effect of oil on natural sediment associated bacteria was minor. Oil significantly lowered the abundance but had no effect on the diversity of fungi in water and sediment. Oil in water form a thin layer on the surface of water, eliminating the light diffusion, and oxygen penetration in marine systems (Bovio et al., 2017). The oil-induced anoxic condition is thus not favored by fungi, nor by aerobic bacteria.

The ratio between an oil-degradation active gene and the 16S rRNA gene copy number was used as an indicator of the biodegradation potential and the oil-contamination level in the environmental samples (Cébron et al., 2008). Cytochrome P450 monooxygenases are important enzymes responsible for the aerobic degradation of hydrocarbons in bacteria, archaea, and eukarya (e.g., fungi and fish) in various environments (Lee and Anderson, 2005; van Beilen et al., 2006; Syed et al., 2013; Fuentes et al., 2014). In the control basins, the higher ratio of P450 gene relative to the 16S rRNA gene copy number in sediment than in water indicates a higher potential for oil degradation in the sediment than in the water in pristine conditions.

Oil significantly increased the portion of oil-degraders that could produce cytochrome P450 monooxygenases in bacterial community in water by 6–7 times. In our study, there was a lag between the oil contamination event until the oil-degrading population peaked at Week 6, suggesting an adaption strategy of oil degraders in the community in response to oil. Oil addition in the surface water may not only inhibit the development of the microbes that are directly involved in aerobic hydrocarbon degradation but also restrict those that are responsible for other ecosystem activities, such as ammonia-oxidizing bacteria, due to competition between species under lowered oxygen concentration condition and high organic matter loading (Wang et al., 2016). As the oil-degrading bacteria may rely on syntrophic bacteria for nutrient and metabolites, the lag might also reveal regained activities of these syntrophic bacteria in the newly established basin environment. However, the copy ratio in sediment was not affected by oil contamination in the sediment, suggesting that the response of the oil-degrading communities was limited by the bioavailability of hydrocarbons.

The qPCR amplification of *alkB* and PAH-RHD GN genes in our samples were not very successful, likely indicating that the P450 or other unstudied oil-degradation enzymatic mechanisms were more important in the studied conditions.

### Community Level Response

In our study, the effect of time on bacterial and fungal community structure was overriding, regardless of the ecosystems. The temporal changes of microbial communities might reflect the microbial adaptation and colonization processes in the basins, regardless of oil contamination. Surface water and sediment represent very different ecosystems, whereas the littoral setting make the two ecosystems connected. These two ecosystems process different physicochemical parameters and mechanisms, which provide divergent substrates and resources for different consortia of microbes. Microbial community structure in quartz sand showed high similarity to those in the surrounding water, regardless of the kingdoms, indicating that a large fraction of the microbes found in the quartz sand were originated from the surrounding water. In connection with the natural sand the bacterial diversity in the water was increased both in the uncontaminated and the contaminated situation.

The significance of oil contamination on microbial communities was dependent on the ecosystems, because sediment was obviously less contaminated than the surface water. The bacterial community was more sensitive than fungal community in responding to oil contamination as the oil-induced bacterial community succession was significant even in the low-oil-contaminated littoral sediment. In water, the response of microbial communities to oil was clearly time-dependent, likely owing to the oil concentration decreasing with time and the composition of oil mixture also changing with time. The successional changes of microbial communities in response to oil contamination have been well characterized in different environments from lab to field scales (Liu et al., 2011; Almeida et al., 2013; Mukherjee et al., 2013; Mikkonen et al., 2014; Yan et al., 2018a, 2020). In our previous survey, oil was often detected in the littoral sediment but rarely detected in the surface water near the oil facility sites presumably contaminated with long-term low oil input (Yan et al., 2018b). The low-molecular-weight hydrocarbon factions are dominant in the water column, whereas the higher-molecular-weight aromatics are preferentially adsorbed by particulate matter and incorporated in the Baltic Sea sediment (Witt, 1995). Together with our results on the microbial diversity and community composition, we believe that the rapid oil degradation occurred in the surface water, whereas the recalcitrant hydrocarbons were mainly retained, sorbed and slowly biodegraded in the littoral sediment with time.

### Identification of Potential Oil-Degraders

Oil-utilizing microorganisms are widely distributed in natural environments (Varjani, 2017). In this study, the majority of bacterial OTUs in the natural sediment were not affected by oil contamination, as oil concentration was low. In the quartz sand sediment, all of the identified oil-responding OTUs were oil-favored. Hydrocarbons can be important C and energy sources. Many of the oil-responding OTUs detected in quartz sand belonged to the same taxonomic groups as those detected in water, although they were not exactly the same OTUs.

In the water, most of the bacterial OTUs that responded to oil contamination showed higher abundance in the presence of oil. The importance of the identified oil-favored OTUs affiliated to Alphaproteobacteria, Gammaproteobacteria, Flavobacteriia and Cytophagia was widely reported to be oil-degraders in diverse oil-polluted environments (Leys et al., 2005; Kostka et al., 2011; Jin et al., 2012; Jurelevicius et al., 2012; Liu and Liu, 2013; Kappell et al., 2014; Lamendella et al., 2014; Abed et al., 2015; Liao et al., 2015). Among Betaproteobacteria, members of Comamonadaceae and Burkholderiaceae were more abundant in the oil-contaminated water than in the control water. Burkholderiaceae are associated with anaerobic benzene and toluene degradation (Van der Zaan et al., 2012; Táncsics et al., 2018). One Comamonadaceae OTU was enriched in oil-contaminated water connected with natural sand, likely revealing its origin from natural sand. This is in line with the finding that Comamonadaceae was found to degrade hydrocarbon mostly in soil environment (Sheng et al., 2016).

In recent years, many hydrocarbon degrading fungal species, for example *Aspergillus* spp., *Penicillium* spp., *Gibellulopsis* spp. and *Candida* spp., have been isolated from contaminated costal seawater (Maddela et al., 2015; Simister et al., 2015; Ameen et al., 2016; Maamar et al., 2020). These fungi are also often found in oil contaminated soil and sediment (Quintella et al., 2019), indicating that the littoral terrestrial system likely serves as the source of inoculants. Miettinen et al. (2019) claims that marine oil degrading fungi are often affected by the Baltic Sea conditions, suggesting that a controlled microcosm is ideal to study the role of these microbes. In this experiment, a number of fungal taxa were found oil-favored in water, most of which were members of Ascomycota. In anaerobic and oligotrophic conditions, certain mitotic fungal taxa belonging to Ascomycota, Basidiomycota, and Zygomycota can possess metabolic adaptations of denitrification, i.e., to utilize nitrate and/or nitrite as an alternative for oxygen (Kurakov et al., 2008). Ascomycota (e.g., *Aspergillus* and *Penicillium*) are able to degrade complex organic compounds e.g., aliphatic and aromatic hydrocarbons in heavily contaminated environments (Wang et al., 2012; Bovio et al., 2017). *Gibellulopsis* spp. (belonging to Sordariomycetes) can use hydrocarbons as sole carbon source (Garzoli et al., 2015; Bovio et al., 2017). In addition, several unclassified fungi were identified more abundant in oil-contaminated water than in the control water, plausibly indicating the great importance of those poorly recognized fungal taxa in oil degradation. Taken together, the abundance increase of those potential indigenous oil-degraders likely suggests that biodegradation was an important oil removal process in the oil-contaminated basins during the 8-week experiment.

## Conclusion

This experimental system combined next generation sequencing and quantitative PCR to investigate the keys players of oil degradation with natural microbiome and thus advanced understanding of the fate of accidental diesel oil spills in Baltic Sea. Time had an overriding effect on microbial succession. Therefore, the duration of the experiment is important to assess the ecological effect of oil in a simulation experiment. The rapid activities of oil removal (e.g., evaporation and degradation) likely occurred mostly in the surface water. Shifts in microbial communities, the trajectories of oil-responding taxa, and the increased ratio of P450:16S rRNA gene copies in oil-contaminated water together suggest that natural attenuation of contamination has occurred from the second Week after the accidental oil spill. The adsorption of the oil by the SOM in the natural sediment might inhibit the diesel oil degradation in a short-term. However, the sediment can function as an important gene bank for supplying the surrounding water with high diversity of oil-degrading communities and further facilitating oil degradation in the long run. The spill of diesel oil into water leads to successive blooms of bacteria belonging to Alphaproteobacteria, Betaproteobacteria, Gammaproteobacteria, Flavobacteriia, and Cytophagia and fungal taxa (mainly belong to Ascomycota). This study revealed an ecological success of bacteria over fungi and archaea in the oil-contaminated environments at the coast of the Gulf of Finland.

## Data Availability Statement

The datasets presented in this study can be found in online repositories. The names of the repository/repositories and accession number(s) can be found in the article/Supplementary Material.

## Author Contributions

MR designed the study jointly with NH. LY, SS, LT, and NH performed the laboratory experiments. LY analyzed the data statistically and wrote the first draft of the manuscript. LY, NH, and MR finalized the manuscript. All the authors contributed to the article and approved the submitted version.

## Conflict of Interest

The authors declare that the research was conducted in the absence of any commercial or financial relationships that could be construed as a potential conflict of interest.
