# Supplementary material for: Simulation of Microbial Response to Accidental Diesel Spills in Basins Containing Brackish Sea Water and Sediment
Source: Front Microbiol. 2020 Dec 23;11:593232. doi: 10.3389/fmicb.2020.593232 (PMC7785775; doi:10.3389/fmicb.2020.593232)
Supplement: Supplementary file 1 [file Data_Sheet_1.PDF]

Supplementary materials

Supplementary Figures

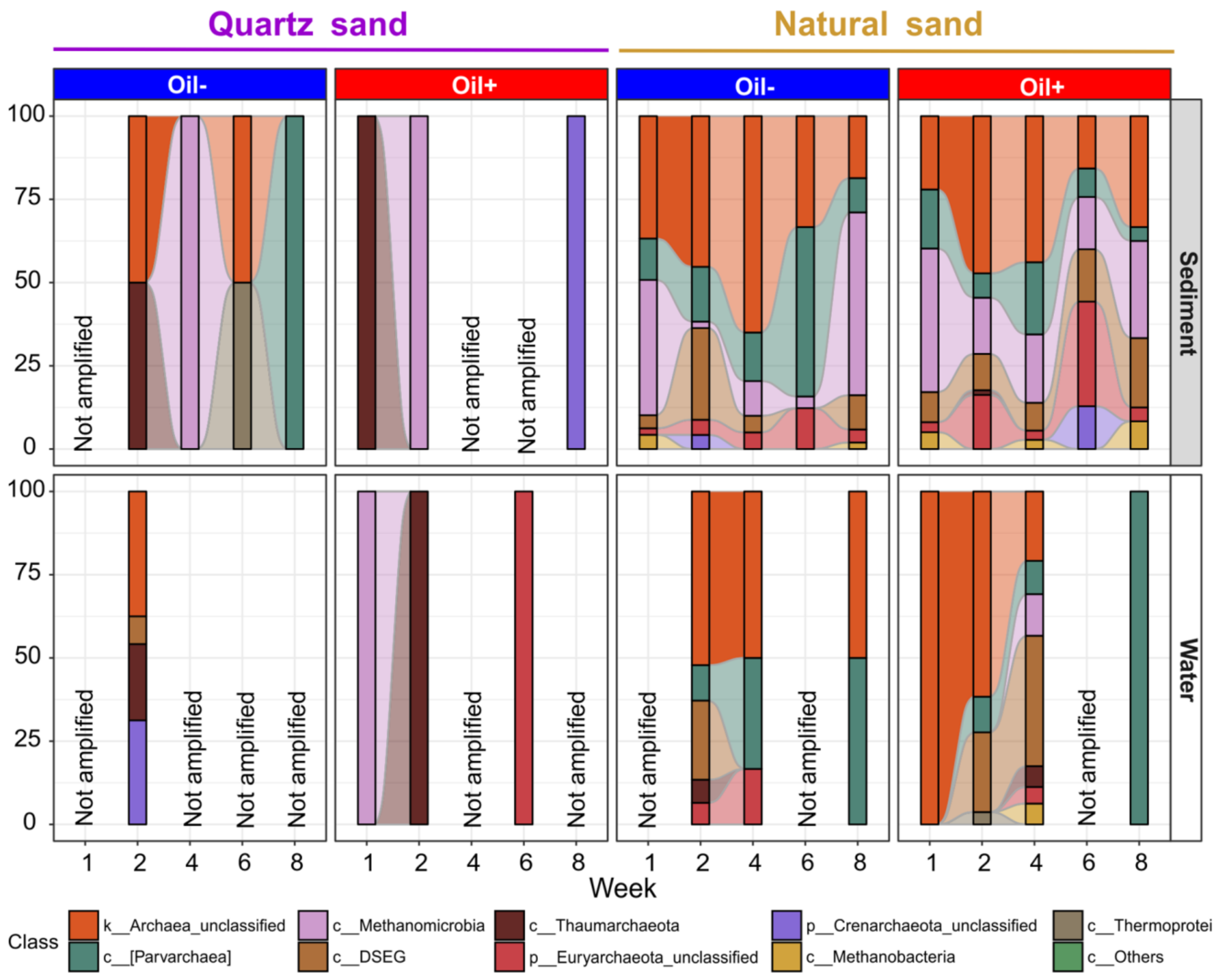

Figure S1. Archaeal community composition in each basin between different ecosystems over time.

Treatment			Oil	pH	DNA	logBac	logFun	logArc	Fun:Bac	logP450	logalkB	logPAH	norm P450	norm alkB	norm PAH
Quartz sand	Week 1	Oil+	6.7	7.6	621	22.3	13.3	16.5	0.01%	8.7	<LOQ	9.7	0.04%	<LOQ	0.000%
		Oil-	<LOQ	7.7	1398	22.6	13.8	16.8	0.02%	9.8	15.4	10,2	0.09%	0.03%	0.000%
	Week 2	Oil+	4.5	7.7	939	18.5	13,5	16.6	0.76%	10.0	14.5	<LOQ	5.60%	1.29%	<LOQ
		Oil-	NA	8.0	1019	22.2	15.5	16.1	0.16%	9.6	16.2	<LOQ	0.13%	0.16%	<LOQ
	Week 4	Oil+	3.3	7.7	285	18.2	11.4	17.2	0.12%	9.8	12.8	<LOQ	6.74%	0.35%	<LOQ
		Oil-	NA	8.0	471	19.4	15.6	15.2	2.17%	9.0	13.8	<LOQ	0.89%	0.46%	<LOQ
	Week 6	Oil+	3.3	8.2	906	17.6	11.3	18.1	0.22%	10.8	14.5	11.5	36.71%	0.42%	0.019%
		Oil-	NA	8.1	294	17.5	12.1	17.7	0.49%	9.7	15.7	<LOQ	8.88%	14.39%	<LOQ
	Week 8	Oil+	0.3	8.2	1228	18.8	11.0	19.4	0.04%	12.6	14.0	11.3	63.27%	0.63%	0.034%
		Oil-	<LOQ	8.1	308	18.2	13.1	16.9	0.68%	8.7	<LOQ	<LOQ	2.01%	<LOQ	<LOQ
Natural sand	Week 1	Oil+	8.8	7.5	1566	22.8	14.1	17.0	0.02%	10.1	<LOQ	9.7	0.08%	<LOQ	0.000%
		Oil-	<LOQ	7.5	1381	23.3	13.8	17.0	0.01%	10.5	15.3	9.8	0.07%	0.03%	0.000%
	Week 2	Oil+	7.8	7.4	1915	19.3	15.1	17.3	1.49%	11.1	15.2	9.2	8.01%	0.81%	0.002%
		Oil-	NA	7.8	1754	22.5	17.3	16.7	0.59%	9.7	15.3	<LOQ	0.08%	0.07%	<LOQ
	Week 4	Oil+	0.6	7.4	1293	20.1	13.0	18.9	0.09%	12.4	14.6	<LOQ	16.25%	0.94%	<LOQ
		Oil-	NA	7.8	1445	20.0	16.2	16.6	8.95%	11.1	<LOQ	10.4	5.04%	<LOQ	0.011%
	Week 6	Oil+	3.7	7.9	867	16.6	12.8	17.4	6.09%	11.3	14.3	<LOQ	343.1%	1.55%	<LOQ
		Oil-	NA	8.0	769	18.9	16.4	18.7	9.63%	12.2	<LOQ	10.7	36.39%	<LOQ	0.029%
	Week 8	Oil+	0.4	8.0	2674	18.6	13.4	18.5	0.81%	12.9	16.7	10.4	173.0%	55.02%	0.01%
		Oil-	<LOQ	8.1	2010	19.4	16.1	19.3	3.98%	12.6	11.7	11.3	33.26%	0.06%	0.047%

High

Low

Non-parametric testing:

	Oil	pH	DNA	logBac	logFun	logArc	Fun:Bac	logP450	logalkB	logPAH	norm P450	norm alkB	norm PAH
Time (Kruskal-Wallis)	***	***	*	ns	**	***	***	**	ns	*	***	**	**
Conta. (Wilcoxon)	***	**	ns	***	***	*	ns	ns	ns	ns	**	**	ns
Sand type (Wilcoxon)	ns	**	***	ns	***	*	*	***	ns	ns	ns	ns	ns
SandConta. (Kruskal-Wallis)	***	***	***	***	***	***	*	***	*	ns	*	*	ns

Figure S2. Heatmap table showing the mean values of the measured environmental and biotic factors between treatments in water. Abbreviations: *Oil* oil concentration (unit: g L^{-1}), *DNA* DNA concentration (unit: ng L^{-1}), *logBac* natural logarithm transformed values of bacterial 16S rDNA copies; *logFun* natural logarithm transformed values of fungal ITS copies; *logArc* natural logarithm transformed values of archaeal 16S rDNA copies; *Fun:Bac* the ratio of fungal ITS copies to bacterial 16S rDNA copies; *logP450* natural logarithm transformed values of P450 copies; *logalkB* natural logarithm transformed values of alkB copies; *logPAH* natural logarithm transformed values of PAH-RHD GN copies; *normP450* P450 copies normalized with bacterial 16S rDNA copies per sample; *normalkB alkB* copies normalized with bacterial 16S rDNA copies per sample; *normPAH* PAH-RHD GN copies normalized with bacterial 16S rDNA copies per sample; *<LOQ* below the limit of quantitation.

Treatment			Oil	TOM	DNA	logBac	logFun	logArc	Fun:Bac	logP450	logalkB	logPAH	norm P450	norm alkB	norm PAH
Quartz sand	Week 1	Oil+	4.6	0.6%	125	11.1	17.5	16.2	0.18%	13.3	13.4	<LOQ	1.52%	1.51%	<LOQ
		Oil-	0.1	0.8%	160	11.6	18.3	17.1	0.25%	13.5	13.9	<LOQ	2.19%	2.87%	<LOQ
	Week 2	Oil+	11.4	0.5%	201	11.4	17.8	17.6	0.18%	14.7	14.4	<LOQ	4.30%	3.22%	<LOQ
		Oil-	NA	0.6%	194	13.8	17.6	17.6	2.34%	14.5	14.1	<LOQ	4.75%	2.97%	<LOQ
	Week 4	Oil+	18.9	0.4%	151	11.5	17.9	17.9	0.16%	14.8	13.4	<LOQ	4.76%	1.31%	<LOQ
		Oil-	NA	0.7%	83	15.0	16.9	16.8	15.21%	13.4	12.4	<LOQ	2.95%	0.67%	<LOQ
	Week 6	Oil+	11.3	0.9%	105	11.4	16.8	17.0	0.54%	14.6	12.6	<LOQ	12.02%	0.61%	<LOQ
		Oil-	NA	0.8%	86	11.7	15.6	16.6	2.13%	14.4	<LOQ	<LOQ	29.04%	<LOQ	<LOQ
	Week 8	Oil+	11.6	0.9%	218	10.6	17.9	18.3	0.07%	16.4	13.3	11.0	24.22%	1.22%	0.129%
		Oil-	<LOQ	1.2%	104	13.2	17.0	16.7	4.11%	14.1	<LOQ	<LOQ	6.88%	<LOQ	<LOQ
Natural sand	Week 1	Oil+	8.2	1.9%	1632	16.5	20.2	19.9	2.46%	17.8	16.1	<LOQ	9.21%	1.95%	<LOQ
		Oil-	0.2	2.0%	1762	16.5	20.2	20.0	3.55%	17.6	16.0	<LOQ	9.37%	2.61%	<LOQ
	Week 2	Oil+	9.1	2.5%	3057	17.2	20.7	20.1	3.10%	18.0	<LOQ	<LOQ	6.65%	<LOQ	<LOQ
		Oil-	NA	1.1%	2603	16.5	20.0	19.5	3.16%	17.1	<LOQ	<LOQ	5.77%	<LOQ	<LOQ
	Week 4	Oil+	5.9	1.9%	2138	16.4	20.6	20.8	1.51%	18.3	16.5	9.8	10.01%	1.98%	0.002%
		Oil-	NA	1.8%	1721	17.2	20.5	20.5	3.90%	18.0	16.4	<LOQ	8.38%	2.21%	<LOQ
	Week 6	Oil+	5.8	2.3%	1874	16.1	20.3	20.9	1.65%	18.1	16.5	11.0	11.36%	1.72%	0.008%
		Oil-	NA	2.1%	2371	17.0	20.2	21.1	4.48%	18.1	16.3	10.2	11.98%	2.13%	0.005%
	Week 8	Oil+	4.3	2.2%	3207	16.0	20.8	20.9	0.92%	18.8	16.7	10.7	14.03%	1.98%	0.004%
		Oil-	<LOQ	2.6%	3701	16.7	20.5	20.3	2.25%	18.5	15.8	10.3	13.38%	0.90%	0.002%

High

Low

Non-parametric testing:

	Oil	TOM	DNA	logBac	logFun	logArc	Fun:Bac	logP450	logalkB	logPAH	normP450	normalkB	normPAH
Time (Kruskal-Wallis)	ns	ns	ns	ns	ns	ns	ns	ns	ns	ns	***	*	ns
Conta. (Wilcoxon)	***	ns	ns	ns	*	ns	***	ns	ns	ns	ns	ns	ns
Sand type (Wilcoxon)	ns	***	***	***	***	***	***	***	***	ns	**	ns	*
SandConta. (Kruskal-Wallis)	***	***	***	***	***	***	***	***	***	ns	*	ns	ns

Figure S3. Heatmap table showing the mean values of the measured environmental and biotic factors between treatments in sediment. Abbreviations: *Oil* oil concentration (unit: g kg^{-1}), TOM total organic matter of sand materials; *DNA* DNA concentration (unit: ng g^{-1}), *logBac* natural logarithm transformed values of bacterial 16S rDNA copies; *logFun* natural logarithm transformed values of fungal ITS copies; *logArc* natural logarithm transformed values of archaeal 16S rDNA copies; *Fun:Bac* the ratio of fungal ITS copies to bacterial 16S rDNA copies; *logP450* natural logarithm transformed values of P450 copies; *logalkB* natural logarithm transformed values of alkB copies; *logPAH* natural logarithm transformed values of PAH-RHD GN copies; *normP450* P450 copies normalized with bacterial 16S rDNA copies per sample; *normalkB* alkB copies normalized with bacterial 16S rDNA copies per sample; *normPAH* PAH-RHD GN copies normalized with bacterial 16S rDNA copies per sample; *<LOQ* below the limit of quantitation.

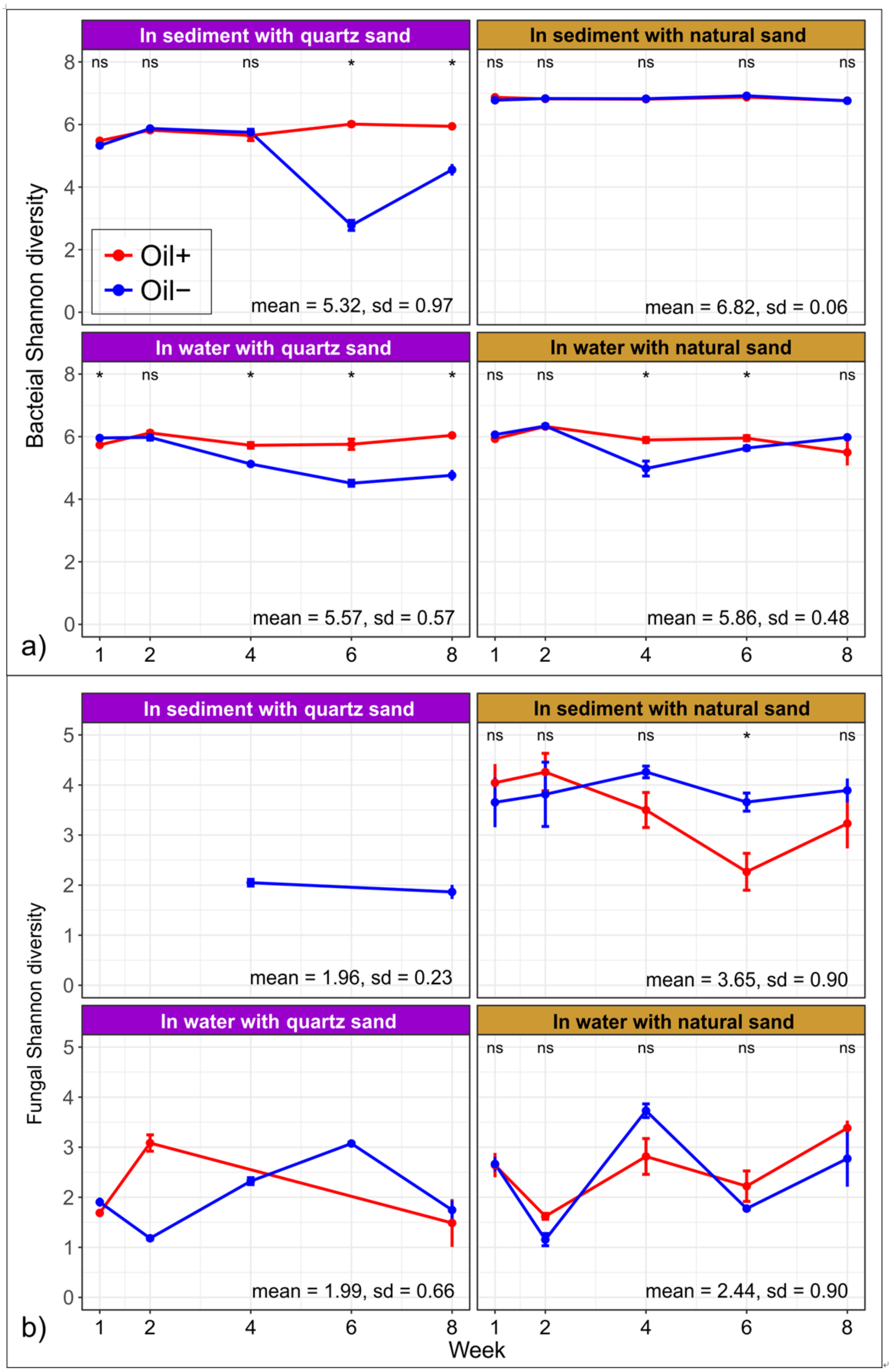

Figure S4. Alpha diversity of bacterial (a) and fungal (b) communities in different ecosystems over time. Statistical results based on one-way Wilcoxon test can be seen in Table 2. Error bars represent standard errors of the mean. Fungal ITS sequences were not amplified well in the quartz sediment

and water connected to quartz sediment in the oil-contaminated basins, so the effect of oil contamination in these basins were not tested.

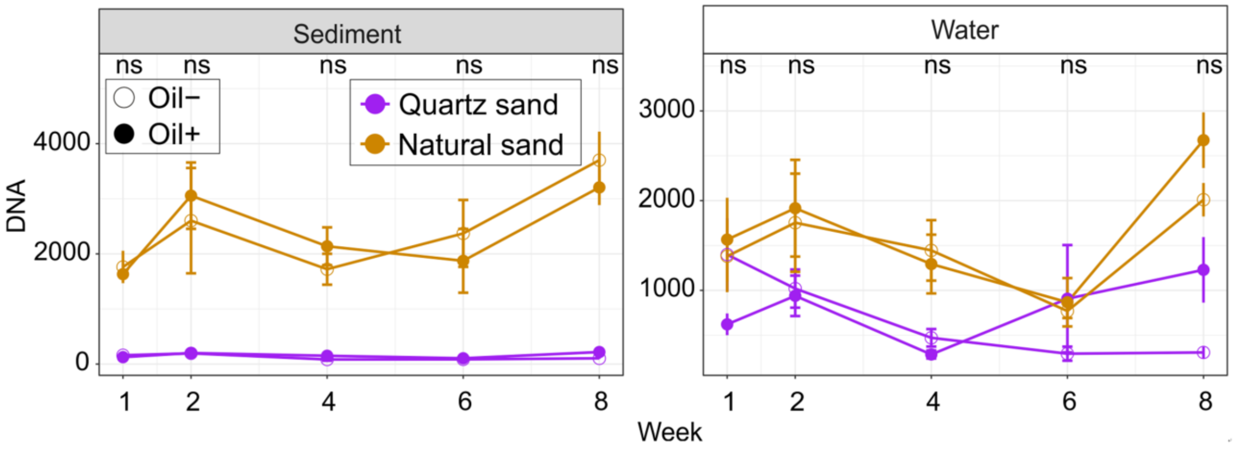

Figure S5. Concentration of extracted genomic DNA (as a proxy of microbial biomass, unit: ng g^{-1} sediment or ng L^{-1} water) in the basins. The effect of oil contamination on the DNA concentration were tested with Wilcoxon test: "***" $p < 0.001$, "**" $p < 0.01$, "*" $p < 0.05$ and "ns" not significant.

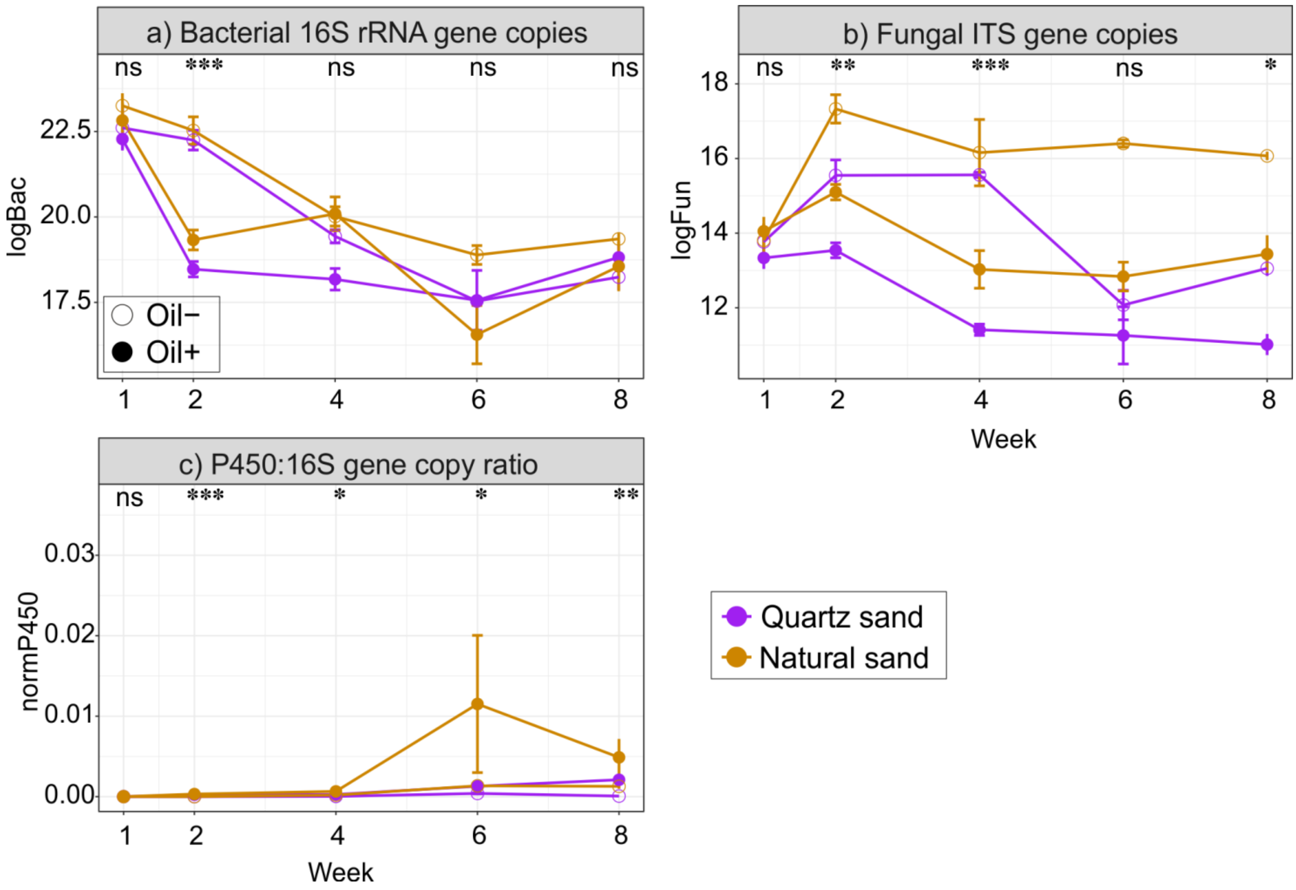

Figure S6. Quantitative analysis of (a) bacterial 16S copies, (b) fungal ITS copies and (c) copy ratio of P450 gene to bacterial 16S rRNA gene in the surface water in the basins over time. Abbreviations and units: logBac natural logarithm transformed values of bacterial 16S rRNA gene copy; logFun natural logarithm transformed values of fungal ITS copies; P450:16S gene copy ratio

absolute copies ratio between P450 and bacterial 16S rRNA gene; All marker genes were measured in copies per gram of oven dry sand or per litter of water, depending on the ecosystems. The effect of oil contamination on the gene copies or the copy ratio was tested with Wilcoxon test: “***” $p < 0.001$, “**” $p < 0.01$, “*” $p < 0.05$ and “ns” not significant.

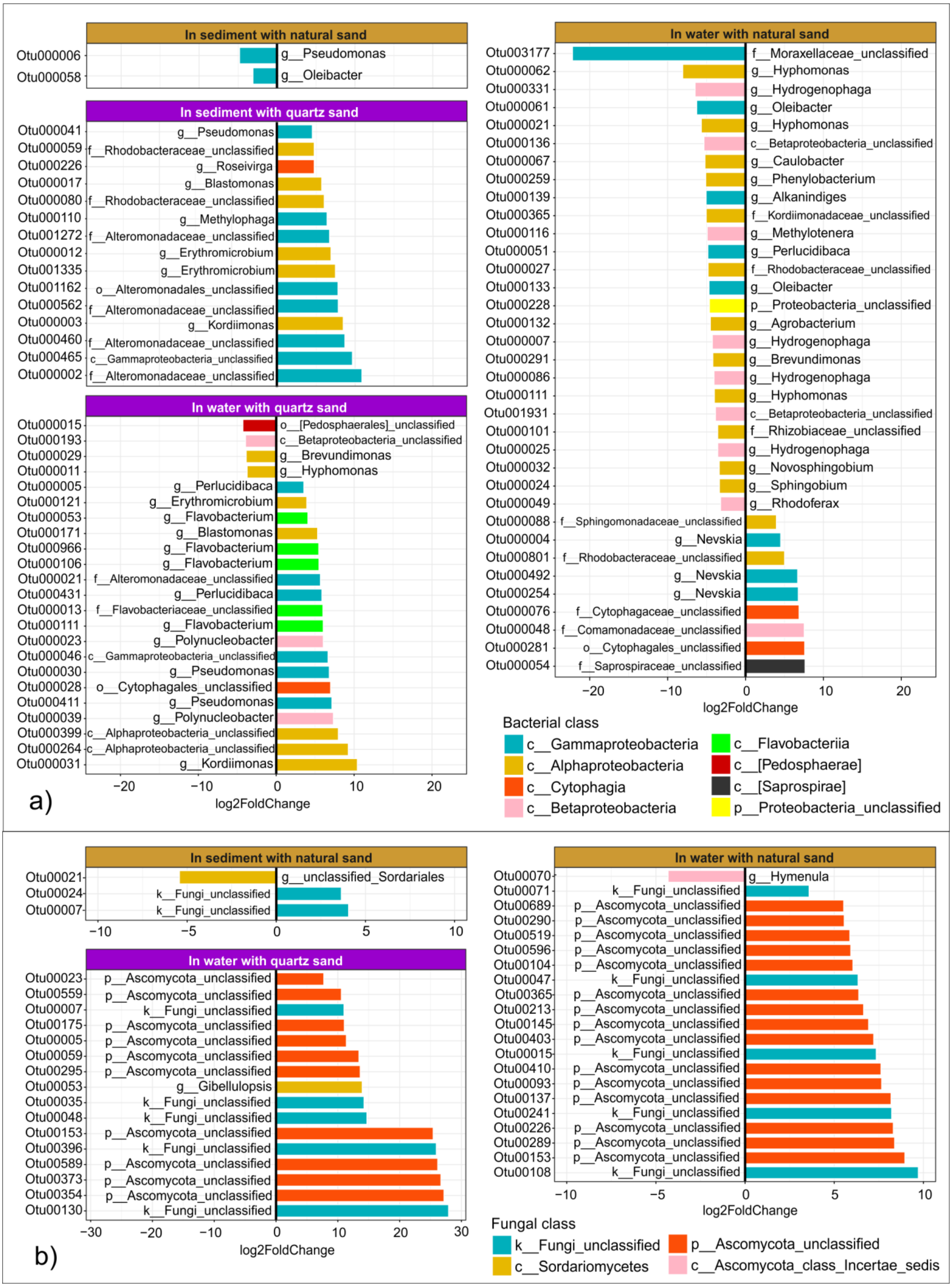

Figure S7. Oil-specific bacterial (a) and fungal OTUs (b) detected by DESeq2. The rare OTUs (relative abundance < 0.05%) were removed prior to DESeq2 analysis (Log2FoldChange > 3, p < 0.001).

Supplementary materials
