# Supplementary material for: Simulation of Microbial Response to Accidental Diesel Spills in Basins Containing Brackish Sea Water and Sediment
Source: Front Microbiol. 2020 Dec 23;11:593232. doi: 10.3389/fmicb.2020.593232 (PMC7785775; doi:10.3389/fmicb.2020.593232)
Supplement: Supplementary file 2 [file Data_Sheet_2.PDF]

Supplementary Tables

Table S1. Physicochemical properties of the seawater and quartz sand materials used in the study

Property	Water	Natural sand	Artificial sand
pH	7.60	5.75	7.00
Cond.	8.94	61	63
Oil	<LOQ	0.17	0.14
Total N	315	-	-
Salinity	4.98	-	-
TOC	10.28	-	-
DM	-	81.36%	75.75%
TOM	-	1.95%	0.76%

Abbreviations: *Cond.* Conductivity (units: mS/m), *Oil* oil concentration (unit: g kg^{-1} sediment or g L^{-1} water), Total N total nitrogen content (unit: µg L^{-1}); *Salinity* water salinity (unit: o/oo), *TOC* total organic carbon in water (unit: mg L^{-1}); *DM* dry matter content; *TOM* total organic matter of sediment materials; *LOQ* limit of quantification.

Table S2. PCR programs for amplicon sequencing

Kingdoms	Reagents	1 x reaction	PCR program:
Bacterial 16S rRNA	H_2O	15.75	1. 98 for 30 sec
	HF Buffer	5.00	2. 98 for 10 sec
	dNTP mix	0.50	3. 51 for 30 sec
	341F (10 µM)	1.25	4. 72 for 15 sec
	806R (10 µM)	1.25	5. 19 more cycles
	Polymerase	0.25	6. 72 for 10 min
	Template	1.00	6. 4 hold
	Total	25.00	
Fungal ITS	H_2O	14.75	1. 98 for 30 sec
	HF Buffer	5.00	2. 98 for 10 sec
	dNTP mix	0.50	3. 57 for 30 sec
	ITS1F (10 µM)	1.25	4. 72 for 15 sec
	ITS2R (10 µM)	1.25	5. 29 more cycles
	Polymerase	0.25	6. 72 for 10 min
	Template	2.00	6. 4 hold
	Total	25.00	
Archaeal 16S rRNA	H_2O	14.75	1. 98 for 30 sec
	HF Buffer	5.00	2. 98 for 10 sec
	dNTP mix	0.50	3. 58 for 30 sec
	344F (10 µM)	1.25	4. 72 for 15 sec
	806R (10 µM)	1.25	5. 29 more cycles

Polymerase	0.25	6. 72 for 10 min
Template	2.00	6. 4 hold
Total	25.00	

The primers used for amplification of the partial bacterial 16S rRNA gene (V3-V4 region) were 341F (5'-CCTACGGGAGGCAGCAG-3') and 806R (5'-GGACTACHVGGGTWTCTAAT-3') (Caporaso et al., 2011; Muyzer et al., 1993). Fungal ITS PCR primers were ITS1F (5'-CTTGGTCATTTAGAGGAAGTAA-3') and ITS2R (5'-GCTGCGTTCTTCATCGATGC-3') (Gardes and Bruns, 1993; White et al., 1990). The primers used for partial archaeal 16S rRNA gene (V3-V4 region) were ARC344F (5'-ACGGGGYGCAGCAGGCGCGA-3') and Arch806R (5'-GGACTACVSGGGTATCTAAT-3') (Raskin et al., 1994; Takai and Horikoshi, 2000).

Table S3. qPCR programs for quantification of bacterial 16S rRNA, fungal ITS and archaeal 16S rRNA genes

Kingdoms	Reagents	1 x reaction	qPCR program:
Bacterial 16S rRNA	H_2O	3.00	1. 95 °C for 15 min
	Master Mix	5.00	2. 94 °C for 10 sec
	pE (10 µM)	0.50	3. 60 °C for 20 sec
	pF (10 µM)	0.50	4. 72 °C for 30 sec
	Template	1.00	5. Plate Read
			6. Go to line 2 for 34 more cycles
			7. Incubate at 95 °C for 10 sec
			8. Melting Curve from 65 °C to 95 °C, increment 0.5°C for 5 sec + plate read
	Total	10.00	END
Fungal ITS	H_2O	3.00	1. 95 °C for 15 min
	Master Mix	5.00	2. 94 °C for 10 sec
	ITS3F (10 µM)	0.50	3. 56 °C for 20 sec
	ITS4R (10 µM)	0.50	4. 72 °C for 30 sec
	Template	1.00	5. Plate Read
			6. Go to line 2 for 34 more cycles
			7. Incubate at 95 °C for 10 sec
			8. Melting Curve from 65 °C to 95°C, increment 0.5 °C for 5 sec + plate read
	Total	10.00	END
Archaeal 16S rRNA	H_2O	3.00	1. 95 °C for 15 min
	Master Mix	5.00	2. 94 °C for 10 sec
	ARCHF1369 (10 µM)	0.50	3. 58 °C for 20 sec
	PROKR1541 (10 µM)	0.50	4. 72 °C for 30 sec
	Template	1.00	5. Plate Read
			6. Go to line 2 for 34 more cycles
			7. Incubate at 95 °C for 10 sec
			8. Melting Curve from 65 °C to 95°C, increment 0.5 °C for 5 sec + plate read
	Total	10.00	END

Master mix used was from DyNAmo HS SYBR Green qPCR Kit (Thermo Scientific, Finland). The primers qPCR used for bacterial 16S rRNA gene were pE (5'- AAA CTC AAA GGA ATT GAC GG -3') and pF (5'- ACG AGC TGA CGA CAG CCA TG -3') (Öqvist *et al.*, 2008). Primers for fungal ITS gene were ITS3F (5'- GCA TCG ATG AAG AAC GCA GC -3') and ITS4R (5'- TCC TCC GCT TAT TGA TAT GC -3') (White *et al.*, 1990). Primers for archaeal 16S rRNA gene were Arch1369F (5'- CGG TGA ATA YGY CCC TGC -3') and Prok1541R (5'- AAG GAG GTC ATC CRG CCG CA -3') (Suzuki *et al.*, 2000). Quality control: All standards were linear, with R^2>0.985, Error <0.25 and Efficiency 80%-110%. The Cq Error between replicates of a sample was always less than 0.5. In the event

that the negative control was positive, samples with Cq < Cq - 7 were considered usable. All samples had a single peak in their melting curves, close to but often at a slightly higher temperature compared to the standards. Samples that did not meet the quality parameters were re-run.

Table S4. qPCR programs for quantification of selected oil-degrading genes

Functional genes	Reagents	1 x reaction	PCR program:
Cytochrome P450	H_2O	6.00	1. 95 for 15 min
	Master Mix	10.00	2. 94 for 10 sec
	P450fw1 (10 µM)	1.00	3. 60 for 20 sec
	P450rv3 (10 µM)	1.00	4. 72 for 20 sec
	Template	2.00	5. Plate Read
			6. Go to line 2 for 34 more cycles
			7. Incubate at 95 for 10 sec
			8. Melting Curve from 65 to 95, increment 0.5C for 5 sec + plate read
	Total	20.00	END
alkB	H_2O	6.00	1. 95 for 15 min
	Master Mix	10.00	2. 94 for 10 sec
	alkBF (10 µM)	1.00	3. 55 for 20 sec
	alkBR (10 µM)	1.00	4. 80 for 20 sec
	Template	2.00	5. Plate Read
			6. Go to line 2 for 34 more cycles
			7. Incubate at 95 for 10 sec
			8. Melting Curve from 65 to 95, increment 0.5C for 5 sec + plate read
	Total	20.00	END
PAH-RHD_{α}GN	H_2O	6.00	1. 95 for 15 min
	Master Mix	10.00	2. 94 for 10 sec
	PAH-RHD_{α} GN F (10 µM)	1.00	3. 57 for 20 sec
	PAH-RHD_{α} GN R (10 µM)	1.00	4. 83 for 20 sec
	Template	2.00	5. Plate Read
			6. Go to line 2 for 34 more cycles
			7. Incubate at 95 for 10 sec
			8. Melting Curve from 65 to 95, increment 0.5C for 5 sec + plate read
	Total	20.00	END

Master mix used was from DyNAmo HS SYBR Green qPCR Kit (Thermo Scientific, Finland). The primers qPCR used for cytochrome P450 monooxygenase gene were P450fw1(5'- GTSGGCGGCAACGACACSAC -3') and P450rv3 (5'-GCASCGGTGGATGCCGAAGCCRAA -3') (van Beilen et al., 2006). The primers qPCR used for alkB gene were alkBF (5'- AACTACATCGAGCACTACGG -3') and alkBR (5'-TGAAGATGTGGTTGCTGTTCC -3') (Powell et al., 2006). The primers qPCR used for PAH-RHD_{α} GN gene were PAH-RHDα GN F (5'- GAGATGCATACCACGTKGGTTGGA -3') and PAH-RHDα GN R (5'- AGCTGTTGTTCGGGAAGAYWGTGCMGTT-3') (Cébron et al., 2008). For quality control, all standards were linear, with $R^2>0.985$, Error <0.25 and Efficiency 80%-110%. The Cq Error between replicates of a sample was always less than 0.5.

In the event that the negative control was positive, samples with Cq < Cq - 7 were considered usable. All samples had a single peak in their melting curves, close to but often at a slightly higher temperature compared to the standards. Samples that did not meet the quality parameters were re-run.
